# Effect of flecainide derivatives on sarcoplasmic reticulum calcium release suggests a lack of direct action on the cardiac ryanodine receptor

**DOI:** 10.1111/bph.13521

**Published:** 2016-06-29

**Authors:** Mark L Bannister, Anita Alvarez‐Laviada, N Lowri Thomas, Sammy A Mason, Sharon Coleman, Christo L du Plessis, Abbygail T Moran, David Neill‐Hall, Hasnah Osman, Mark C Bagley, Kenneth T MacLeod, Christopher H George, Alan J Williams

**Affiliations:** ^1^ Wales Heart Research InstituteCardiff University School of MedicineCardiffUK; ^2^ Myocardial Function Section, National Heart and Lung InstituteImperial College LondonLondonUK; ^3^ Department of Chemistry, School of Life SciencesUniversity of SussexBrightonUK; ^4^ School of Chemical SciencesUniversiti Sains MalaysiaPenangMalaysia

## Abstract

**Background and Purpose:**

Flecainide is a use‐dependent blocker of cardiac Na^+^ channels. Mechanistic analysis of this block showed that the cationic form of flecainide enters the cytosolic vestibule of the open Na^+^ channel. Flecainide is also effective in the treatment of catecholaminergic polymorphic ventricular tachycardia but, in this condition, its mechanism of action is contentious. We investigated how flecainide derivatives influence Ca^2^^+^‐release from the sarcoplasmic reticulum through the ryanodine receptor channel (RyR2) and whether this correlates with their effectiveness as blockers of Na^+^ and/or RyR2 channels.

**Experimental Approach:**

We compared the ability of fully charged (QX‐FL) and neutral (NU‐FL) derivatives of flecainide to block individual recombinant human RyR2 channels incorporated into planar phospholipid bilayers, and their effects on the properties of Ca^2^^+^ sparks in intact adult rat cardiac myocytes.

**Key Results:**

Both QX‐FL and NU‐FL were partial blockers of the non‐physiological cytosolic to luminal flux of cations through RyR2 channels but were significantly less effective than flecainide. None of the compounds influenced the physiologically relevant luminal to cytosol cation flux through RyR2 channels. Intracellular flecainide or QX‐FL, but not NU‐FL, reduced Ca^2^^+^ spark frequency.

**Conclusions and Implications:**

Given its inability to block physiologically relevant cation flux through RyR2 channels, and its lack of efficacy in blocking the cytosolic‐to‐luminal current, the effect of QX‐FL on Ca^2^^+^ sparks is likely, by analogy with flecainide, to result from Na^+^ channel block. Our data reveal important differences in the interaction of flecainide with sites in the cytosolic vestibules of Na^+^ and RyR2 channels.

**Abbreviations**
CPVT: catecholaminergic polymorphic ventricular tachycardia
NCX: Na^+^/ Ca^2^^+^ exchanger
RyR2: cardiac ryanodine receptor
SR: sarcoplasmic reticulum

## Tables of Links

**Table:** 

**TARGETS**
**Voltage‐gated ion channels** *a*
Na_v_1.5 channels
**Ligand‐gated ion channels** *b*
RyR2 channels
**Transporters** *c*
NCX, Na^+^/ Ca^2+^ exchanger

**LIGANDS**
Flecainide

These Tables list key protein targets and ligands in this article which are hyperlinked to corresponding entries in http://www.guidetopharmacology.org, the common portal for data from the IUPHAR/BPS Guide to PHARMACOLOGY (Southan *et al*., 2016) and are permanently archived in the Concise Guide to PHARMACOLOGY 2015/16 (^*a*,*b*,c^Alexander *et al*., 2015a, 2015b, 2015c).

## Introduction

Flecainide is a well‐characterized blocker of sarcolemmal Na^+^ channels (Liu *et al*., 2002; Liu *et al*., 2003) and K^+^ channels (Follmer and Colatsky, 1990; Paul *et al*., 2002). In addition, flecainide has been shown to be effective in the treatment of catecholaminergic polymorphic ventricular tachycardia (CPVT), and its action has been attributed to an ability to reduce inappropriate release of Ca^2^^+^ from the sarcoplasmic reticulum (SR) by directly blocking open cardiac ryanodine receptor (RyR2) channels (Watanabe *et al*., 2009; Hilliard *et al*., 2010). However, in previous studies, we have established that while flecainide can act as a partial blocker of cytosolic to luminal flux of cations in RyR2 channels, it is unable to influence the physiologically relevant flux of cations from the SR lumen to the cytosol that occurs during Ca^2^^+^ release. As a consequence, we have concluded that the mechanism of action of flecainide in CPVT depends on its ability to block Na^+^ channels and does not involve a direct action on RyR2 channels (Bannister *et al*., 2015). This issue is important to resolve because disruption of intracellular calcium homeostasis is also linked to dysfunction in skeletal muscle (muscular dystrophy and malignant hyperthermia), smooth muscle (asthma), brain (stroke and Alzheimer's disease) and the endocrine system (type 2 diabetes mellitus). Defective RyR‐mediated Ca^2^^+^ release thus is involved in diseases of global prevalence and importance, and RyR is therefore an important therapeutic target (e.g. Mackrill, 2010; Santulli and Marks, 2015).

Block of Na^+^ flux into the cell by flecainide results from entry of the ligand from the cytosol and interaction with a site in the cytosolic vestibule of the Na^+^ channel (Liu *et al*., 2003). Similarly, flecainide interacts with an equivalent site within the cytosolic vestibule of RyR2 channels and gains access to this site from the cytosol (Mehra *et al*., 2014; Bannister *et al*., 2015). Flecainide bound in RyR2 channels influences cation flux from the cytosolic side of the channel to the SR lumen; a current that may contribute to charge compensation during Ca^2^^+^ release (Gillespie and Fill, 2008), but is rapidly displaced by cations moving in the physiologically relevant direction (from the SR lumen to the cytosol).

**Figure 1:**
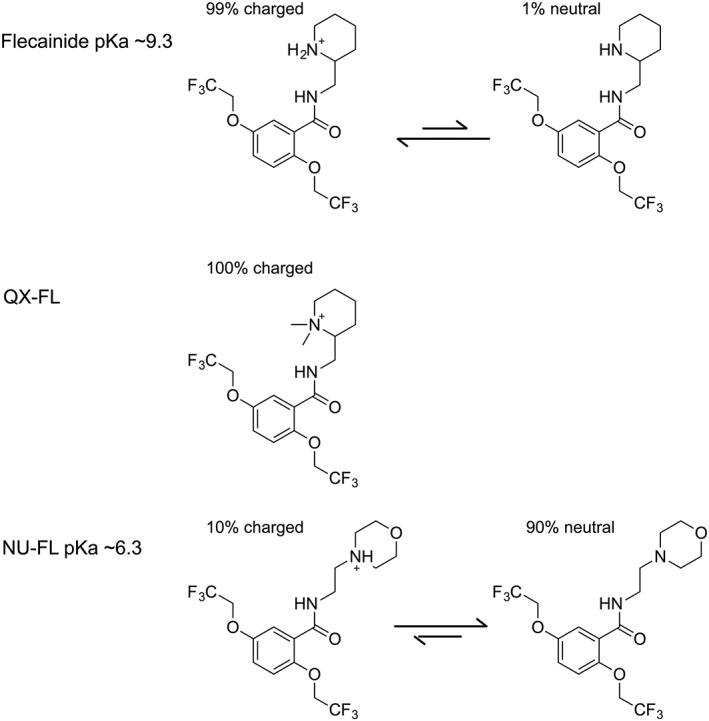
Chemical structures of flecainide, QX‐FL and NU‐FL. At pH 7.4, flecainide (pKa 9.3) is >99% charged; NU‐FL (pKa 6.4) is >90% neutral. QX‐FL is permanently charged.

Given the very different abilities of flecainide to block the equivalent, physiologically relevant, flux of cations in the Na^+^ and RyR2 channels, it is important to establish the corresponding mechanisms governing interaction. Important information on the molecular characteristics underlying Na^+^ channel block were obtained using derivatives of flecainide with similar structural features but differing net charge (Liu *et al*., 2003). As highlighted in Figure 1, at physiological pH, 100% of QX‐FL will be cationic while 90% of NU‐FL will be neutral. Investigations of Na^+^ channel block by flecainide, QX‐FL and NU‐FL established that block results from the interaction of the cationic form of the molecule with the Na^+^ channel (Liu *et al*., 2003). Flecainide has also been shown to block hERG channels at physiologically relevant concentrations (Paul *et al*., 2002), and QX‐FL and NU‐FL have been used to demonstrate that, as is the case in the Na^+^ channel, hERG block requires entry of a cationic blocking molecule from the cell interior (Melgari *et al*., 2015). In the present study, we have used QX‐FL and NU‐FL to establish the mechanisms underlying the interaction of flecainide with RyR2 channels.

Our investigations demonstrated that the structural and chemical features governing flecainide interaction with Na^+^ channels and RyR2 channels were different. While ligand charge was the primary determinant of blocking ability in Na^+^ channels, small structural changes severely reduced the ability of flecainide to block the potential charge‐compensating, (cytosolic to SR lumen), flux of cations through RyR2 channels during Ca^2^^+^ release from the SR. Consequently, the flecainide derivatives give us new tools with which to test the mechanism of action of flecainide in the regulation of Ca^2^^+^ release from the SR in CPVT. Both flecainide and QX‐FL (applied inside the cell) are effective blockers of sarcolemmal Na^+^ channels. Data presented here demonstrate that while intracellular flecainide might produce some reduction in a potential RyR2 channel‐mediated cation counter‐current during Ca^2^^+^ release from the SR, QX‐FL will not. Therefore, intracellular QX‐FL is a Na^+^ channel‐specific ligand, and we have compared its action with those of flecainide and NU‐FL on the properties of Ca^2^^+^ sparks in intact adult rat cardiac myocytes.

## Methods

### Cell culture and transfection

HEK293 cells (ECACC, Salisbury, UK) were cultured in Dulbecco's modified‐Eagle medium supplemented with 10%(v/v) fetal bovine serum, 2 mM glutamine and 100 μg · mL^−1^ penicillin/streptomycin (LifeTech, Paisley, UK). Cells were incubated at 37°C, 5% CO_2_ and 80–90% humidity at a density of 2 × 10^6^ per 100 mm plate (60 cm^2^) 24 h prior to transfection with pcDNA3/eGFP‐hRyR2 (6 μg per 1 × 10^6^ cells). After overnight incubation, transfected cells were treated with sodium butyrate (2 mM) for a further 24 h before assessment of expression (by eGFP visualization), harvesting by centrifugation (500 x *g* AllegraR, Beckman) and storage at −80°C.

### Purification of recombinant hRyR2 channels

Frozen cell pellets (typically ~50 × 10^6^ cells) were lysed on ice in a hypo‐osmotic buffer (20 mM Tris–HCl, 5 mM EDTA; pH 7.4) containing protease inhibitor cocktail (Roche), by passing them 20 times through a 23 G needle. Unbroken cells and nuclei were removed by low‐speed centrifugation (1500 x g, 10 min, 4°C, AllegraR, Beckman), and the resulting lysate was subjected to a high‐speed centrifugation (100,000 x *g*, 90 min, 4°C, Optima L‐90 K, Beckman) to collect the microsomal membranes. Solubilization of these membranes was carried out (at a concentration of 2.5 mg · mL^−1^) in a solution containing 1 M NaCl, 0.15 mM CaCl_2_, 0.1 mM EGTA, 25 mM PIPES, 0.6% (w/v) CHAPS and 0.3% (w/v) phospatidylcholine, with protease inhibitor cocktail (Sigma) at 4°C. Insoluble material was removed by centrifugation (15,000 x *g*, 1 h at 4°C) and the supernatant loaded onto a 5–30% (w/v) continuous sucrose gradient. Fractions containing channel proteins were collected after 18 h centrifugation at 4°C and stored at −80°C until use.

### Conditions for recording single hRyR2 channels

Single hRyR2 channels were incorporated into bilayers formed using a suspension of phosphatidylethanolamine (Avanti Polar Lipids, Alabaster, AL, USA) in n‐decane (35 mg · mL^−1^). Bilayers were formed in a solution containing 610 mM KCl, 20 mM HEPES (pH 7.4) in both (*cis* (0.5 mL) and *trans* (1 mL) chambers. Channel incorporation from the *cis* chamber was facilitated by the introduction of an osmotic gradient (using 200 μL 3 M KCl). On stirring, hRyR2 channels incorporate in a fixed orientation such that the *cis* chamber corresponds to the cytosolic side of the channel and the *trans* chamber to the luminal side (Sitsapesan and Williams, 1994; Bannister *et al*., 2015). After channel incorporation, symmetrical ionic conditions were re‐instated by perfusion of the *cis* chamber with a 610 mM KCl, 20 mM HEPES (pH 7.4) solution. All experiments were carried out at room temperature (20–22°C). The effects of flecainide, QX‐FL and NU‐FL were determined after addition of the drug to either *cis* or *trans* chambers at concentrations indicated in the text. We optimized the quantification of block by using conditions that maximize the open duration of the channel, that is, high permeant ion concentration (610 mM K^+^) in the presence of 20 μM EMD 41000, a RyR2 channel agonist shown previously to act via the caffeine‐binding site (McGarry and Williams, 1994).

### Analysis of single‐channel recordings

**Figure 2:**
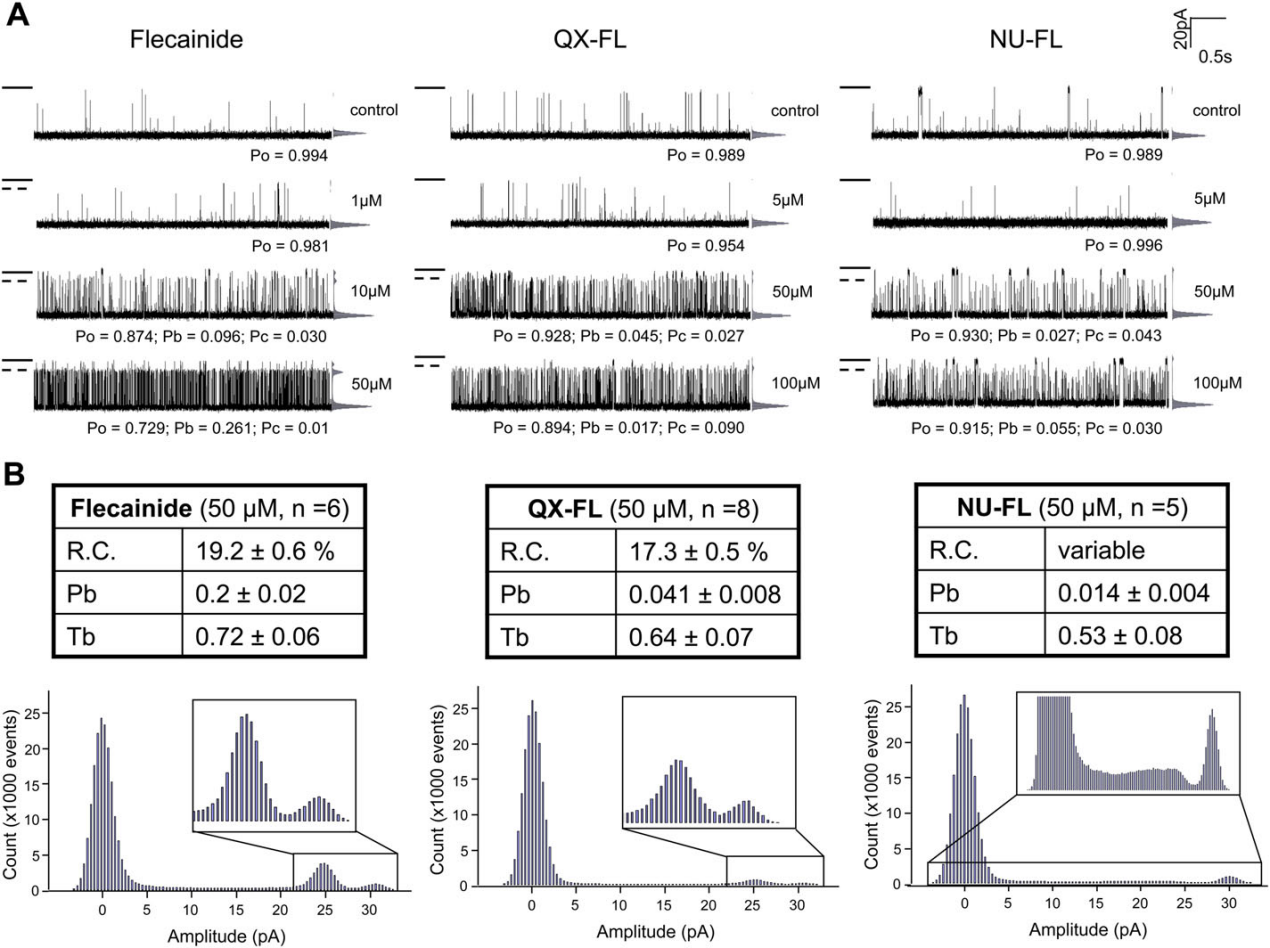
Open channel block of voltage‐driven cytosolic to luminal cation flux through hRyR2 channels. (A) Representative single channel traces recorded at +40 mV in symmetrical 610 mM KCl. Openings are downwards from the closed level (black line). Channels are fully activated with 20 μM EMD 41000. The increase in blocking events (marked with a dotted line) with increasing concentration of flecainide, QX‐FL or NU‐FL is shown in the frequency amplitude histograms and is manifested as a decrease in Po and increase in Pb (B) Expanded frequency amplitude histograms demonstrating the nature of the blocked states, tables above show the mean residual current (R.C.), Pb and Tb achieved with 50 μM of each drug. *n* is given in each instance.

**Figure 3:**
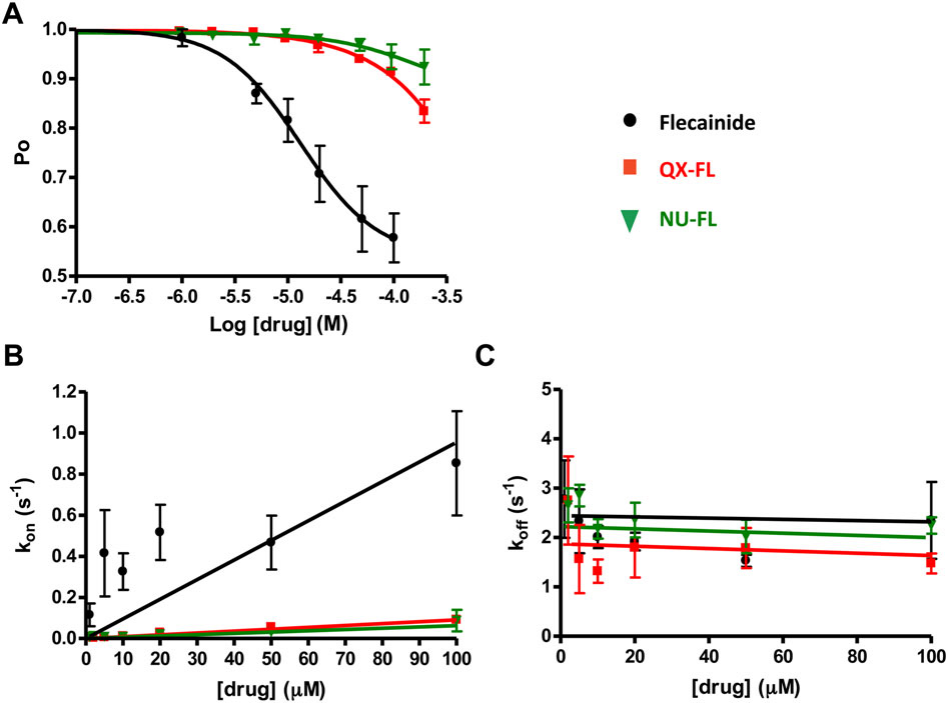
QX‐FL and NU‐FL are less potent blockers of the cytosolic to luminal current through hRyR2 channels compared with flecainide. (A) Decrease in Po at +40 mV observed with increasing concentrations of flecainide, QX‐FL or NU‐FL with corresponding (B) rates of association (*K*_on_, fitted through the origin) and (C) dissociation (*K*_off_). All data points are from *n* = 6 separate experiments.

**Figure 4:**
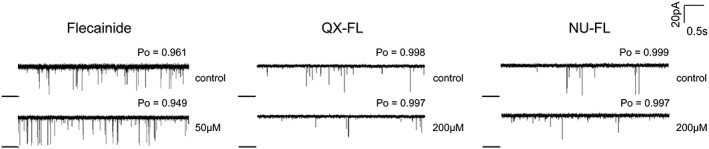
Neither flecainide nor its derivatives block voltage driven luminal to cytosolic cation flux through hRyR2 channels. Representative single channel traces, recorded at −40 mV (luminal to cytosolic current) in the presence of cytosolic ligand (activated with 20 μM EMD41000). Openings are upwards from the closed level (black line); no blocking events or significant changes in Po were observed (*n* = 6).

**Figure 5:**
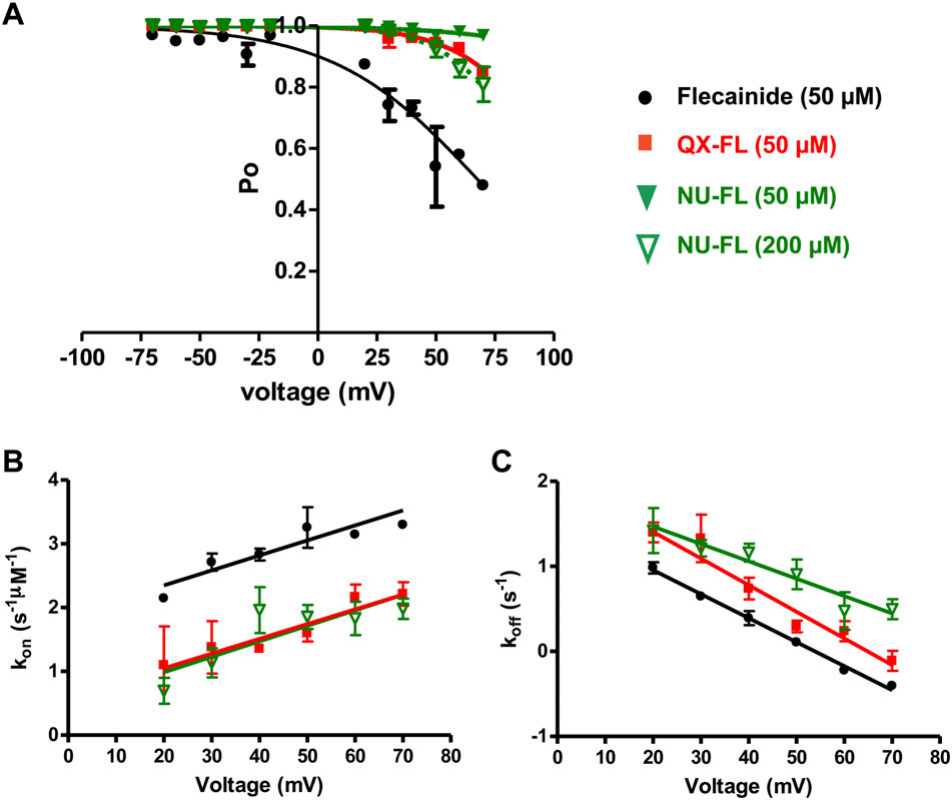
Voltage dependence of block. (A) Plot of Po versus voltage for hRyR2 channels in the presence of 50 μM cytosolic blocker (flecainide *n* = 11, QX‐FL *n* = 8, NU‐FL *n* = 5) determined at holding potentials between ±70 mV. A higher concentration of NU‐FL (200 μM, *n* = 5) is required to see voltage dependence. Dose‐normalized rates of (B) association (K_on_) and (C) dissociation (K_off_) are both voltage‐dependent for flecainide, QX‐FL (each at 50 μM) and NU‐FL (200 μM).

Single‐channel currents were low pass filtered at 5 kHz with an eight‐pole Bessel filter then digitized at 20 kHz with a PCI‐6036E AD board (National Instruments, Austin, TX, USA). acquire 5.0.1 (Bruxton, Seattle, WA, USA) was used for viewing and acquisition of the single channel traces. Data analysis was carried out using qub v2.0.0.13 (www.qub.buffalo.edu). Single‐channel traces of 2–3 min (containing >3000 events) were idealized using the Segmental K‐means algorithm (Qin and Li, 2004) based on hidden Markov models, and a dead time of 75–120 μs was imposed. In traces where substate block was detected (by evaluation of the amplitude histogram), idealization was carried out using a three state [closed (C) ↔ open (O) ↔ blocked (B)] scheme. qub can accurately distinguish between blocked and closed levels and idealization using this scheme resulted in the calculation of mean amplitudes, open (Po), blocked (Pb) and closed (Pc) probabilities and mean open (To), blocked (Tb) and closed (Tc) times. In all other instances where block was not observed, a two‐state (C↔O) scheme was used for idealization, which yielded amplitude, Po, To and Tc as previously described (Mukherjee *et al*., 2012). Po values are fitted using non‐linear regression. Rates of association (*K*_on_) and disassociation (*K*_off_) were calculated as the reciprocal of To and Tb, respectively, for each drug concentration and holding potential and fitted using linear regression (through the origin when calculating *K*_on_ for drug concentration only). Due to the large difference in voltage‐dependence of block by flecainide, QX‐FL and NU‐FL, *K*_on_ and *K*_off_ are plotted in a dose‐normalized manner. Although different example traces are used in Figures 2 and 4, the mean data for flecainide in Figures 3 and 5 are the same as those published in Bannister *et al*. (2015). These data serve as controls against which the actions of QX‐FL and NU‐FL are compared.

### Animals

All animal care and experimental procedures complied with the UK Home Office Animals (Scientific Procedures) Act 1986, which conforms to the Guide for the Care and Use of Laboratory Animals published by the US National Institutes of Health (NIH publication No. 85–23, revised 1996) and approved by the Local Animal Ethics committee (Imperial College Animal Welfare and Ethical Review Committee). Rats were killed by cervical dislocation following exposure to 5% isoflurane until the righting reflex was lost. All procedures involving animals are reported according to the ARRIVE guidelines (Kilkenny *et al*., 2010; McGrath & Lilley, 2015).

### Cardiomyocyte isolation

Ventricular myocytes from healthy adult male Sprague Dawley rats (Charles River, UK, 250 −350 g) were enzymically isolated as previously described (Sato *et al*., 2005). Briefly, hearts were rapidly dissected and then perfused retrogradely using the Langendorff apparatus. Hearts were perfused sequentially with low calcium and a collagenase (Worthington) solution. Ventricles were cut into small pieces and gently minced with a Pasteur pipette. The cell suspension was filtered, and cardiomyocytes were allowed to settle by gravity.

### Electrophysiological recordings combined with calcium imaging

To assess the effects of intracellularly applied drug molecules on the spatial and temporal dynamics of Ca^2^^+^ sparks, confocal microscopy was combined with the whole‐cell voltage clamp recordings. Dual experiments were performed on adult rat ventricular myocytes at room temperature (20–22°C). Cells were superfused with an external physiological solution of the following composition (in mM): 137 NaCl, 10 HEPES, 10 glucose, 6 KCl, 2 MgCl_2,_ 1 CaCl_2_ (pH adjusted to 7.4 with NaOH). Borosilicate microelectrodes were pulled to give a resistance between 2.5 and 3.5 MΩ. Vehicle, flecainide (Sigma) or its analogues QX‐FL and NU‐FL (all at 5 μM) were introduced into the cell cytosol via the patch pipette. Pipettes were filled with an intracellular solution containing either drug or vehicle in addition to membrane‐impermeant dye Fluo‐3 pentapotassium salt (Invitrogen) of the following composition (in mM): 100 K‐aspartate, 15 KCl, 10 HEPES, 5 KH_2_PO_4_, 5 Mg‐ATP, 5 EGTA, 0.75 MgCl_2_, 0.12 CaCl_2_, 0.04 Fluo‐3‐5 K^+^ salt (pH adjusted to 7.2 with 2 M KOH).

After establishing a whole‐cell configuration, vehicle‐ or drug‐containing pipette solution was allowed to equilibrate with the cytosol for 10–15 min. To facilitate steady‐state SR calcium‐loading, cells underwent a series of depolarising steps of 200 ms duration to +30 mV applied from a holding potential of −80 mV at a frequency of 3 Hz for 30 s. Ca^2^^+^ sparks were captured following cessation of voltage clamp stimulation. Electrophysiological recordings were carried out using an Axopatch‐1D amplifier (Axon Instruments, Wokingham, Berkshire, UK) and a Digidata1322A acquisition system (Axon Instruments). Series resistance and whole‐cell capacitance were electronically compensated. Live cell calcium imaging was performed using a laser scanning confocal system (BioRad Microscience Ltd., Hemel Hempstead, Hertfordshire, UK) and an inverted microscope (Nikon Eclipse TE300, Nikon UK Ltd, Kingston Upon Thames, Surrey, UK). Ca^2^^+^ imaging data were acquired and proc‐ Q13 essed using LaserSharp2000 (Biorad) and ImageJ (National Institutes of Health, Bethesda, MD, USA) softwares respectively.

### Data and statistical analysis

Data and statistical analysis comply with the recommendations on experimental design and analysis in pharmacology (Curtis *et al*., 2015). As the cardiac myocytes were prepared from healthy adult male Sprague Dawley rats, there was no requirement for randomization and blinding at this stage. For experiments using cardiac myocytes under voltage clamp conditions, after an initial assessment of Ca^2^^+^ handling in control (drug‐naïve) cells, the inclusion of flecainide, QX‐FL or NU‐FL in the patch pipette was randomly assigned. Data analysis was performed by operators blinded to the drugs under test.

In all experiments, data subjected to statistical analysis are from at least five independent values and are reported as mean ± SEM. The exact value of *n* in each dataset is given in each instance in the corresponding figure legends. Data were tested for normality using the D'Agostino and Pearson omnibus test, and normally and non‐normally distributed data were tested using unpaired Student's *t*‐test or Mann–Whitney test respectively. Data were considered significant if *P* < 0.05. All statistical analysis was performed using PRISM 6.0 software (GraphPad Software Inc., La Jolla, CA, USA).

### Materials

QX‐FL was synthesized as described (Liu *et al*., 2003) while NU‐FL was synthesized using the novel procedure described in Supporting Information. The characterization of use‐dependent block of voltage gated Na^+^ channels by the synthesized QX‐FL, and NU‐FL used in this study is also described in Supporting Information.

## Results

The structures of flecainide, QX‐FL and NU‐FL, together with the proportion of each molecule present as cationic or neutral species at pH 7.4, are shown in Figure 1.

Previous work (Bannister *et al*., 2015) has established that flecainide, present at the cytosolic face of the RyR2 channel, can enter the cytosolic vestibule of the channel and interact with residues in the helices lining this portion of the pore‐forming region. When bound, flecainide introduces a physical and/or electrostatic barrier to the movement of cations from the cytosolic to the luminal side of the channel. The affinity of interaction of flecainide at this site is relatively weak, and bound flecainide is destabilized by movement of cations in the physiologically relevant, luminal to cytosolic direction. In this report, we have examined the influence of QX‐FL and NU‐FL on cation translocation in individual recombinant hRyR2 channels reconstituted into planar phospholipid bilayers and compared these effects with those of flecainide.

The single channel traces in Figure 2(A) show that like flecainide, its derivatives QX‐FL and NU‐FL, present in the solution at the cytosolic face of the channel, are open‐channel blockers of hRyR2 when net cation current is driven cytosolic to luminal by applying a holding potential of +40 mV across the bilayer. The increase in occurrence of blocking events with increasing concentration is evident in the traces themselves and is summarised in the accompanying frequency amplitude histograms. As is the case with flecainide, QX‐FL and NU‐FL do not fully occlude the hRyR2 channel pore, and residual current continues to flow with the blocking molecule bound. The expanded frequency amplitude histograms (Figure 2(B)) highlight a small but significant difference (*P* < 0.05) in the residual current of the blocked states produced by flecainide (19.2 ± 0.6%) and QX‐FL (17.3 ± 0.5%). For NU‐FL, the residual current in the blocked state shows considerable variation.

In all cases, block was manifested as a decrease in Po (Figure 3(A)). The *K*D for flecainide is 13.14 ± 1.89 μM (*n* = 6 channels). The fully charged and neutral analogues block considerably less effectively, and their *K*Ds cannot be calculated from this plot. More quantitative information can be obtained from the rates of association (*K*_on_) and dissociation (*K*_off_) for flecainide, QX‐FL and NU‐FL (*n* = 6 channels) shown in Figures 3(B) and (C). Whilst no significant differences exist between the dissociation rates of the three compounds, rates of flecainide association are significantly higher than those of either QX‐FL or NU‐FL (all in nM^−1^ · s^−1^: flecainide: 9.54 ± 2.20, QX‐FL: 0.91 ± 0.03, NU‐FL: 0.62 ± 0.05; *P* < 0.05).

We next examined whether cytosolic QX‐FL or NU‐FL was able to influence the physiologically relevant flux of cations through hRyR2. This was carried out by reversing the holding potential across the bilayer to −40 mV and so driving net K^+^ current in the luminal to the cytosolic direction (Figure 4). These experiments demonstrate that, as previously shown for flecainide (Bannister *et al*., 2015), neither cytosolic QX‐FL nor NU‐FL is able to influence the physiologically relevant, luminal to cytosolic flux of cations through the hRyR2 channel.

We have extended this investigation by monitoring the effects of varying holding potential on the ability of all three forms of flecainide to block K^+^ flux through the open hRyR2 channel. Figure 5A shows the variation in block caused by 50 μM cytosolic flecainide, QX‐FL and NU‐FL at holding potentials between ±70 mV. Under these conditions, the probability of block for both flecainide and QX‐FL is dependent on potential; being more pronounced as the potential is made increasingly positive. Voltage dependence of block by the predominantly neutral NU‐FL at 50 μM is very weak, but is more pronounced when its association rate is increased on raising the concentration to 200 μM (Figure 5A). These data confirm that for all three compounds block is observed only when net K^+^ flux is in the cytosolic to luminal direction. No block is seen at negative holding potentials when net flux is luminal to cytosolic. In all cases, both rates of blocker association (Figure 5B) and dissociation (Figure 5C) are influenced by transmembrane potential.

These characterizations establish that both QX‐FL and NU‐FL, present in the solution at the cytosolic face of hRyR2 channels are open channel blockers of the non‐physiological flux of cations but are significantly less effective than flecainide. None of the compounds block the physiologically relevant flux of cations when present at the cytosolic face of the channel.

**Figure 6:**
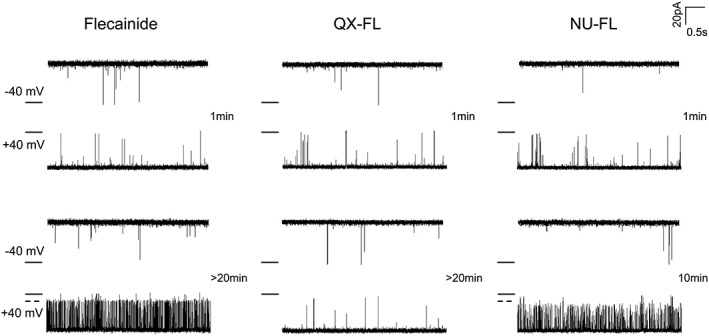
Addition of flecainide or its derivatives to the luminal face of channels. Representative traces of single RyR2 channels recorded at +40 mV before and after the addition of high concentrations of blocker (top panel). QX‐FL does not cross the bilayer and cannot access the channel cytosolic vestibule. Flecainide and NU‐FL are able to equilibrate across the membrane and block the cytosolic to luminal current only (lower panel). Data were obtained from *n* = 3 independent experiments and were not subjected to statistical analysis.

Figure 6 shows the effect of adding each of the blockers to the luminal face of the channel. Under these conditions, no blocking events are seen either at a holding potential of −40 mV, when net cation flux is in the physiologically relevant luminal to cytosolic direction, or initially at +40 mV when net flux is cytosolic to luminal (top panel). These experiments demonstrate that no sites of interaction for flecainide blocking molecules are present at the luminal side of the hRyR2 channel. If a net cytosolic to luminal cation flux is maintained at +40 mV in the presence of luminal flecainide and NU‐FL, brief‐blocking events are apparent after several minutes, and open‐channel block is well established after 10–20 min (lower panel). The neutral forms of these compounds are able to cross the membrane, passing into the solution in the cytosolic chamber and re‐equilibrate here with the cationic form (Figure 1). Only then are the cationic species able to access the cytosolic vestibule of the channel and partly block cytosolic to luminal cation flux. Luminal fully charged QX‐FL cannot cross the bilayer or pass through hRyR2 and, as a consequence, has no access to blocking sites in the cytosolic vestibule.

The data presented to this point indicate that neither QX‐FL nor NU‐FL are as effective as blockers of cytosolic to luminal K^+^ flux through the RyR2 channel as flecainide and, consequently, would be unable to inhibit any contribution that this current might make to RyR2 channel‐mediated charge compensation during Ca^2^^+^ release from the SR. Previous investigations have established that, when present in the cytosol, both flecainide and QX‐FL are potent use‐dependent blockers (UDBs) of sarcolemmal Na^+^ channels (Liu *et al*., 2003). Their very different ability to block a potential charge compensating current through RyR2 means that intracellular QX‐FL is a powerful tool for establishing the relative contribution of Na^+^ channel block, and a potential action on RyR2 channels, to flecainide's inhibition of inappropriate RyR2 channel‐mediated Ca^2^^+^ release in CPVT.

**Figure 7:**
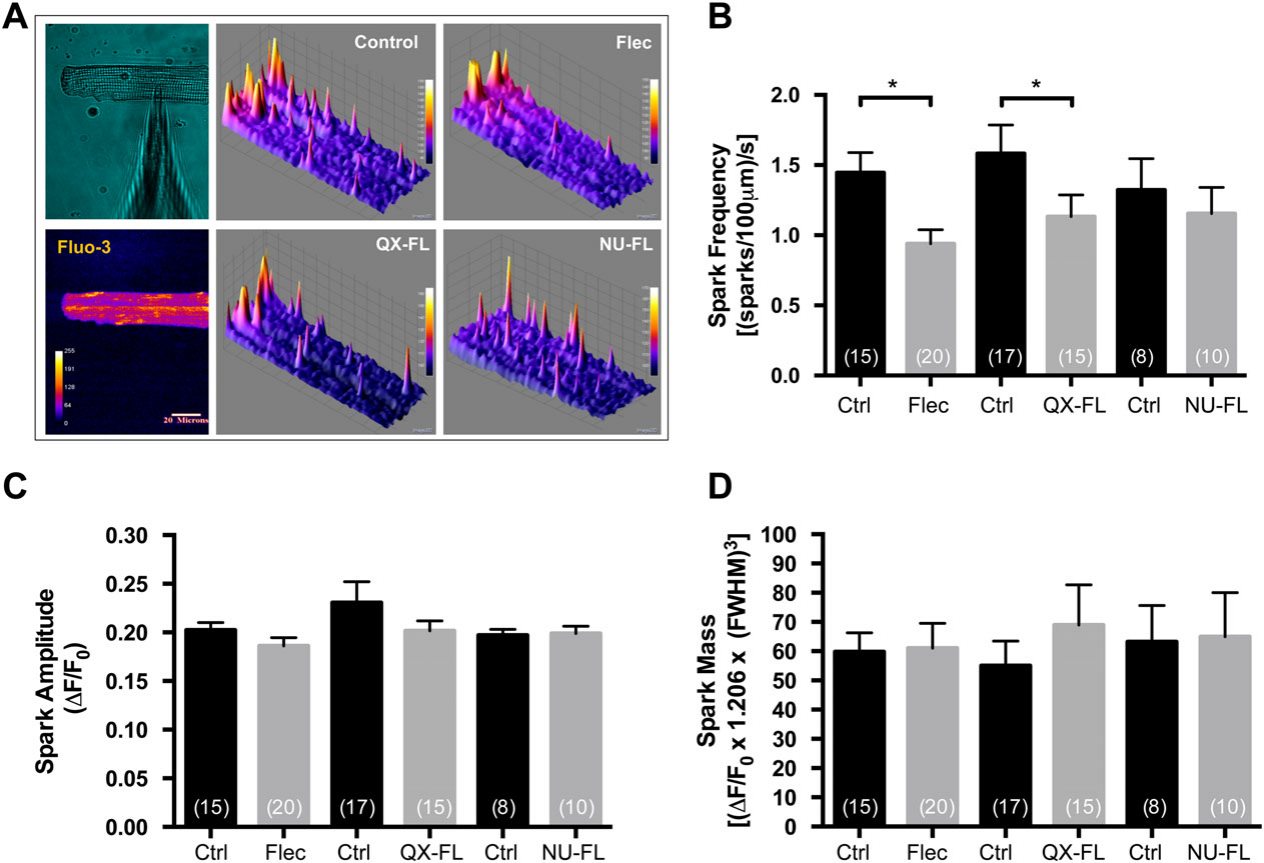
Effects of intracellular application of flecainide and its analogues on Ca^2^^+^ spark parameters. (A) 3D topological view of the spatial and temporal properties of Ca^2^^+^ sparks under voltage clamp stimulation. Cells were dialysed via patch pipette (10–12 min) with either vehicle control solution containing Fluo‐3‐5 K^+^ salt, or with flecainide, QX‐FL or NU‐FL (5 μM). Myocytes were stimulated via depolarization from −80 to +30 mV (3 Hz, 30 s) and spontaneous sparks recorded during a subsequent 20 s quiescent period. (B‐D): The effect of intracellular dialysis of flecainide, QX‐FL or NU‐FL on mean Ca^2^^+^ spark frequency (B), amplitude (C) and mass (D). The number of cardiac myocytes used in each instance is given in parentheses and they were isolated from several different hearts (flecainide *n* = 10/11, QX‐FL *n* = 10, NU‐FL *n* = 5; Supporting Information Table). *, *P* < 0.05; significantly different from the corresponding control (untreated) cells in each group.

Towards this end, we examined the effects of intracellular flecainide and its derivatives on Ca^2^^+^ regulation in rat ventricular myocytes. To measure Ca^2^^+^ sparks, intracellular buffering was adjusted with EGTA to prevent the occurrence of Ca^2^^+^ waves. Lowering the Ca^2^^+^‐buffering capacity of the internal solution to 0.4 mM EGTA gave optimal conditions for selectively targeting SR‐mediated spark activity. Before Ca^2^^+^ spark measurement, myocytes from the control and drug treated group were voltage‐clamp stimulated for 30 s at 3 Hz from a resting membrane potential to +30 mV in order to bring the SR [Ca^2^^+^] to a steady‐state level. Intracellular diastolic [Ca^2^^+^] was calculated to be 77 nM. The effects of intracellular application of flecainide, QX‐FL and NU‐FL on Ca^2^^+^ spark parameters are shown in Figure 7. A 3D topological view of the spatial and temporal properties of Ca^2^^+^ sparks under voltage clamp stimulation is presented in Figure 7A. Flecainide and QX‐FL were equally effective at reducing the frequency of Ca^2^^+^ sparks (Figure 7B), while NU‐FL had no effect on this parameter. None of the compounds had a significant effect on spark amplitude (Figure 7C) or mass (Figure 7D). A detailed analysis of the influence of flecainide, QX‐FL and NU‐FL on Ca^2^^+^ spark parameters is presented in a table in the Supplemental Material.

## Discussion

Flecainide is a well‐characterized UDB of Na^+^ channels (Liu *et al*., 2002; Liu *et al*., 2003). More recently, flecainide has emerged as an effective therapeutic agent for the treatment of CPVT both in combination with conventional β‐adrenoceptor blockade (van der Werf *et al*., 2011; van der Werf *et al*., 2012; Watanabe *et al*., 2013) and as a monotherapy (Napolitano, 2016; Padfield *et al*., 2016).

Given its effectiveness as a Na^+^ channel blocker, it is difficult to envisage a therapeutic action for flecainide that does not involve this target. However, its primary action in CPVT is considered (or has been suggested) to be on RyR2 channels, with the proposal that flecainide blocks the open channel and hence reduces, or prevents, inappropriate RyR2 channel‐mediated release of Ca^2^^+^ from the SR (Hilliard *et al*., 2010).

However, recent work from our group (Bannister *et al*., 2015) has established that while flecainide does interact, with relatively low affinity, with a site within the cytosolic cavity of RyR2 channels, and, when bound, can reduce the flux of monovalent cations in the cytosolic to SR luminal direction through the channel; bound flecainide is displaced by cation flux in the luminal to cytosol direction (i.e. the flux corresponding to the physiologically relevant movement of Ca^2^^+^ from the SR to the cytosol, to initiate contraction during excitation‐contraction coupling). Given this, the only feasible mechanism by which direct interaction of flecainide with RyR2 channels could influence SR Ca^2^^+^ release is by reducing a monovalent cation, charge‐compensating, counter‐current through the channel (Bannister *et al*., 2015).

**Figure 8:**
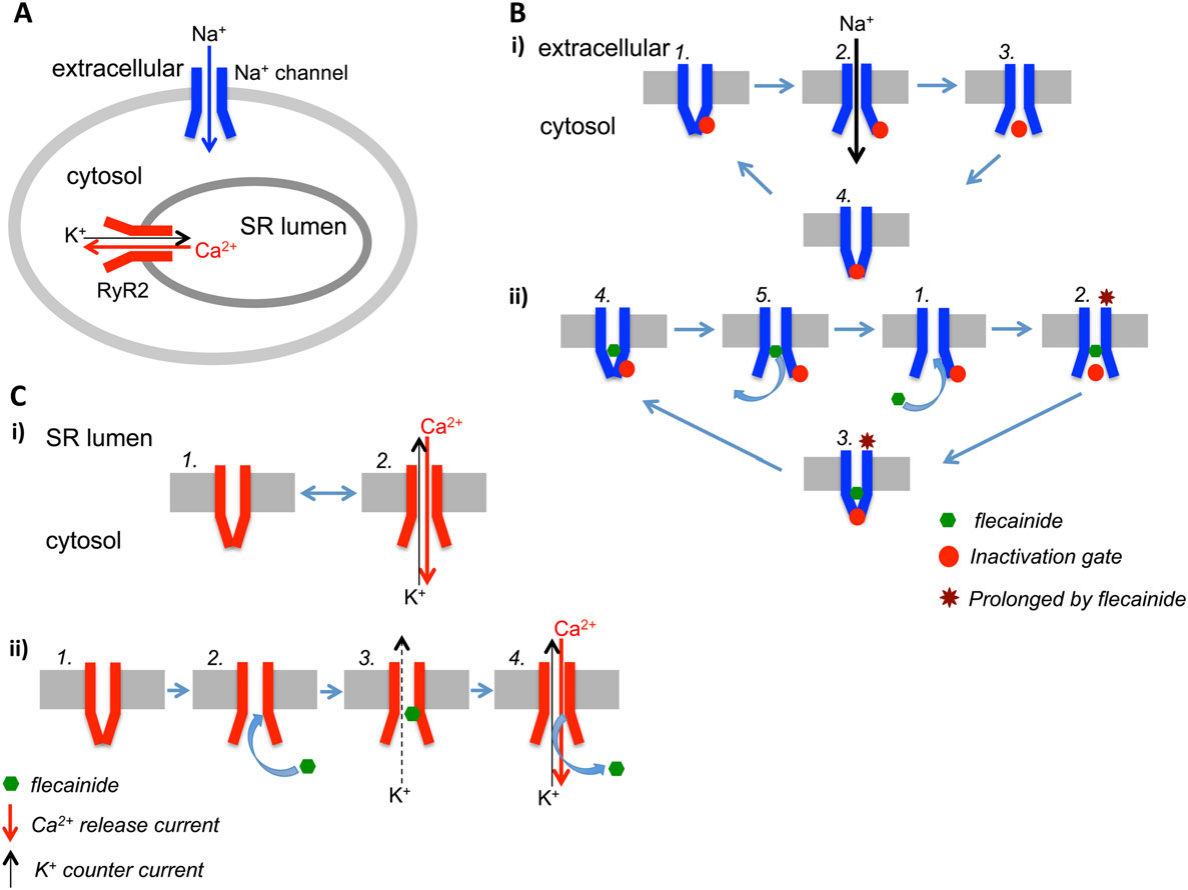
Flecainide inhibits the physiologically relevant cation flux through Na^+^ channels but does not block Ca^2^^+^ release through RyR2 channels. Diagrams showing the orientation of Na^+^ and RyR2 channels in the cardiac myocyte and the consequences of flecainide interaction. (A) The topology of sarcolemmal Na^+^ and sarcoplasmic reticulum RyR2 channels is equivalent. Cation fluxes are indicated by arrows (Na^+^ influx in blue, Ca^2^^+^ release in red and a charge‐compensating K^+^ flux into the SR in black). (B) (i) Simplified scheme for Na^+^ channel gating in the absence of flecainide. 1 – closed; 2 – open; 3 – inactivated; 4 – closed (inactivated). (ii) Simplified scheme for Na^+^ channel gating in the presence of flecainide. 1 – flecainide enters the open channel from the cytosol; 2 and 3 – flecainide prolongs the inactivated and closed (inactivated) conformations; 4 – closed; 5. – flecainide leaves the open channel. (C) (i) Simplified scheme for RyR2 channel gating in the absence of flecainide. 1 – closed; 2 – open allowing Ca^2^^+^ release from the SR (red arrow) and a potential, charge compensating, cytosolic to luminal K^+^ counter current (black arrow). (ii) Simplified scheme for RyR2 channel gating in the presence of flecainide. 1 – closed; 2 – flecainide enters the cytosolic vestibule from the cytosol; 3 – bound flecainide partly blocks the potential, charge‐compensating, cytosolic to luminal K^+^ counter current (dotted black arrow); 4 – bound flecainide is displaced by the physiologically relevant flux of Ca^2^^+^ during release from the sarcoplasmic reticulum.

While casting doubt on the therapeutic significance of a direct action of flecainide on RyR2 channels, this observation does raise an important mechanistic issue. In the cardiac myocyte Na^+^ channels and RyR2 channels are located in different membrane systems. However, their orientation, the direction of physiologically relevant cation flux through the channels, the access route of flecainide to its binding site and the location of the binding site are all equivalent (Figure 8A). Given this equivalence, why then is flecainide a therapeutically relevant blocker of the Na^+^ channel but not of RyR2 channels?

The factors governing flecainide's blocking interaction in the Na^+^ channel cytosolic cavity were revealed by studying the blocking efficiency of flecainide derivatives with similar three‐dimensional structure but differing net charge (Figure 1) (Liu *et al*., 2003). We have used these derivatives in the current investigation to gain new insights into the contribution of structural and charge characteristics to the ability of flecainide to interact with RyR2 channels and to block a potential charge‐compensating, cytosolic to luminal, monovalent cation flux.

Our data demonstrate that (although with dramatically lower affinity), as is the case for flecainide, both QX‐FL and NU‐FL are concentration‐ and voltage‐dependent blockers of a potential charge compensating, monovalent cation flux through RyR2 channels. All three ligands interact within the cytosolic vestibule of the channel pore and gain access to this site from the cytosolic side of the channel. When bound, the ligands do not fully occlude the RyR2 channel pore, rather, they create a steric and/or electrostatic barrier that limits cytosolic to luminal cation flux. The different residual currents seen, with the blocking molecules bound, indicate that the magnitude of the barrier is dependent upon characteristics of the blocking molecule.

Detailed investigation of the effects of flecainide, QX‐FL and NU‐FL on Na^+^ channels established that the efficacy of these ligands as blockers was determined, primarily, by net charge rather than the structural features of the molecule (Liu *et al*., 2003). The data presented here establish that this is not the mechanism that governs the blocking interaction with RyR2 channels. Under the experimental conditions used in our experiments, the proportion of flecainide and QX‐FL molecules that will be positively charged is essentially the same (99% for flecainide and 100% for QX‐FL). However, QX‐FL is a dramatically less effective blocker of the potential charge‐compensating, cytosolic to luminal, flux of K^+^ through RyR2 channels. This observation demonstrates that factors other than net charge contribute to the ability of flecainide to block cytosolic to luminal cation flux in RyR2 channels. This conclusion is strengthened by the observation that NU‐FL (~10% cationic) is only slightly less efficient as a blocker of RyR2 channels than QX‐FL. No significant difference exists in the rate of dissociation of the three blocking molecules from RyR2 channels and the variation in blocking efficiency of flecainide and its derivatives arise from differences in their rates of association with the channel, with this rate, in comparison with flecainide, approximately 10‐fold and 15‐fold lower for QX‐FL and NU‐FL respectively. What characteristics of the molecules underlie this variation?

Previous work from our group has established that a wide range of large monovalent and polyvalent cations are concentration‐ and voltage‐dependent blockers of cytosolic to luminal cation flux through the open RyR2 channel (Tinker *et al*., 1992a, 1992b; Tinker and Williams, 1993; Mead and Williams, 2004; Mason *et al*., 2012). Given this, it is logical to conclude that the open channel block reported here arises from the interaction of the cationic component of flecainide, QX‐FL and NU‐FL, with a site within the cytosolic vestibule of RyR2 channels. Therefore the difference in the absolute concentration of cationic species in flecainide and NU‐FL could be a contributing factor to the differing abilities of these molecules to block RyR2 channels. However, as the absolute concentrations of cationic species in flecainide and QX‐FL are essentially the same, other molecular characteristics must contribute to the difference in the efficacy of these blockers.

In Na^+^ channels, local anaesthetic antiarrhythmics, including flecainide, are stabilized within the cytosolic vestibule by interactions with two aromatic residues separated by two turns on the S6 pore‐lining helix of repeat IV (Ragsdale *et al*., 1994; Ragsdale *et al*., 1996), and high affinity binding is believed to involve cation‐π interactions between these aromatic residues and amine groups of the blockers (Ahern *et al*., 2008). In RyR2 channels, cation‐π interactions are unlikely to underpin flecainide block because there are no aromatic residues in the proposed binding region (the lower cytosolic vestibule of the channel) (Bannister *et al*., 2015). The variation in blocking efficiency of flecainide and its derivatives suggests that the capacity of the molecules to act as hydrogen bond acceptors or donors may be important in determining their ability to bind in the RyR2 channel cytosolic cavity. Considering functional group differences, the secondary amine flecainide can form two hydrogen bonds with the piperidine ring as its conjugate acid, whereas NU‐FL, a tertiary amine, can form only one. QX‐FL, as a quaternary compound, has no hydrogen bonding capacity in that region. Small structural differences among flecainide, QX‐FL and NU‐FL (Figure 1) may also contribute to the likelihood of interaction with RyR2 channels. The bonding around the ring nitrogen in QX‐FL and the cationic forms of flecainide and NU‐FL is not identical, and steric encumbrance around this atom may result in reduced efficacy.

Crucially, the consequence of flecainide interaction with the Na^+^ channel differs from that with RyR2 channels in one very important way; although, access to the flecainide binding site in Na^+^ channels requires the channel to be open, once bound, flecainide interacts preferentially with the inactivated closed conformation of the channel, prolonging this state, thereby limiting the channel's availability for further activation (Liu *et al*., 2002). This limited egress of flecainide from the Na^+^ channel facilitates the accumulation of block (or use‐dependence) with repetitive depolarizations of the cell (Liu *et al*., 2003). Therefore, in Na^+^ channels, flecainide does not merely act as a blocking substrate, it also effectively alters the gating of the channel. Gating of RyR2 channels involves transitions between open or closed states but, unlike the Na^+^ channel, the RyR2 channel does not need to pass through an inactivated state, so in these channels, flecainide acts simply as an open‐channel partial blocker of cytosolic to luminal cation flux, and use‐dependent block does not occur. The absence of an effect on gating, and the low affinity with which flecainide binds within the cytosolic vestibule of RyR2 channels, means that bound flecainide is readily displaced by the physiologically relevant, luminal to cytosolic, flux of Ca^2^^+^ from the SR during E‐C coupling (Bannister *et al*., 2015). The mechanisms governing flecainide interaction in Na^+^ and RyR2 channels are summarized in Figure 8(B) and (C) respectively.

The data presented here and in Bannister *et al*. (2015) establish that the only way in which flecainide could act directly on RyR2 channels to inhibit Ca^2^^+^ release would be via a very small reduction of an RyR2 channel‐mediated counter current. The demonstration that QX‐FL is essentially without effect on this current identifies this ligand as a tool with which to examine if counter current inhibition contributes, in any way, to flecainide's therapeutic action in CPVT. Intracellular QX‐FL and flecainide are both highly effective blockers of the sarcolemmal Na^+^ channel (Liu *et al*., 2003, Supporting Information Figure S2), while even the small, potential, effect of flecainide on RyR2 channel‐mediated counter‐current will be absent with intracellular QX‐FL. Consistent with the conclusion that the action of flecainide on Ca^2^^+^ handling in cardiac myocytes results solely from inhibition of the Na^+^ channel, intracellular flecainide and QX‐FL have equivalent effects on Ca^2^^+^ sparks. NU‐FL, a considerably less efficient Na^+^ channel blocker, has no effect on Ca^2^^+^ handling.

In intact cardiomyocytes, whole‐cell patch clamp‐mediated intracellular application of 5 μM flecainide, and QX‐FL, but not NU‐FL, decreased spark frequency with no effect on spark amplitude and mass (Figure 7 and Supporting Information Table). These data suggest that it is the charged form of flecainide that mediates this effect and corroborate and extend previous findings that flecainide suppresses Ca^2^^+^ spark frequency (Sikkel *et al*., 2013a). Taken together with the present investigations that confirmed a lack of effect of these concentrations of flecainide and QX‐FL on the physiologically relevant luminal‐to‐cytoplasmic ion flux through RyR2 channels and, in the case of QX‐FL, any inhibition of a potential charge‐compensating cytosolic‐to‐luminal K^+^ flux (Figures 4, 5, 6), these data support the conclusion that the only mode of action underpinning flecainide's efficacy in CPVT patients is the inhibition of I_Na_ (Liu *et al*., 2011; Sikkel *et al*., 2013a; Bannister *et al*., 2015). In addition, our demonstration that QX‐FL is a Na^+^ channel specific ligand, yet has identical effects to flecainide on Ca^2^^+^ spark parameters (Supplementary Table) reinforces this conclusion.

Sikkel *et al*. (2013a) showed that the flecainide‐mediated reduction in Na^+^ influx into the cardiomyocyte can, via the enhancement of Ca^2^^+^ efflux through the Na^+^/ Ca^2^^+^ exchanger (NCX), decrease [Ca^2^^+^]_i_ in the vicinity of the RyR2 channels and thus reduce the frequency of spontaneous SR Ca^2^^+^ release events. These authors also showed that this was a class effect of I_Na_ blockers, and moreover, flecainide caused no reduction in SR Ca^2^^+^ leak when I_Na_ was eliminated by altering the holding potential via a voltage clamp. By amalgamating these findings with their existent hypothesis that flecainide's mechanism of action was dependent on direct effects on I_Na_ and through direct interaction with RyR2 channels, Steele and co‐workers proposed a ‘triple mode’ of flecainide action, namely, via its direct effects on I_Na_ and RyR2 channels and an indirect effect on NCX (Steele *et al*., 2013). The present investigations, which corroborates previous work by Bannister and colleagues (Bannister *et al*., 2015), present the unequivocal demonstration that flecainide does not have a direct effect on luminal‐to‐cytosolic Ca^2^^+^ flux through RyR2 channels, and fundamentally challenges reports that flecainide efficacy in RyR2 channel‐ and CSQ‐mutation linked CPVT patients is due to an additive effect of I_Na_ inhibition and a direct block of RyR2 channels (Watanabe *et al*., 2009; Hilliard *et al*., 2010; Galimberti and Knollmann, 2011; Hwang *et al*., 2011; Lee *et al*., 2012). Further corroborating the lack of direct effect of flecainide on RyR2 channel‐mediated Ca^2^^+^ release, Supporting Information Figure S3 shows the persistence of RyR2 channel‐dependent spontaneous Ca^2^^+^ release events in Na_v_1.5‐null HEK (HEK) in the presence of flecainide (5 μM).

The contention that flecainide is less effective in inhibiting RyR2 channels under circumstances that promote higher spark frequency (i.e. conditions of Ca^2^^+^ overload) (Steele *et al*., 2013; Sikkel *et al*., 2013b) is not applicable here because the present studies in intact control cells are characterized by comparable Ca^2^^+^ spark frequencies with those reported by Hilliard *et al*. (≈1.5 versus 0.8, respectively), suggesting similar SR Ca^2^^+^ loading. Adding to the confusion, the same group has recently described the increased potency of flecainide in suppressing intracellular Ca^2^^+^ waves in permeabilized cardiomyocytes following manoeuvres that increase Ca^2^^+^ spark frequencies (Savio‐Galimberti and Knollmann, 2015).

We have established that the principal action of flecainide in CPVT is not via a direct interaction with RyR2 channels. Our data support a model of flecainide action in which Na^+^‐dependent modulation of intracellular Ca^2^^+^ handling attenuates RyR2 channel dysfunction in CPVT. These findings are crucial as they will contribute to the rational design of improved therapies and prevent the clinical misuse of flecainide in the treatment of phenotypically similar but mechanistically distinct arrhythmias.

## Author contributions

A.J.W., M.L.B., N.L.T., A.A.L. and C.H.G. designed the study; M.L.B. and N.L.T. performed single channel experiments; A.A.L. performed myocyte Ca^2^^+^ imaging experiments; S.C. and C.H.G. created the H‐Flp/Na_v_1.5 cells; S.A.M. characterized Na^+^ channel function; C.d.P., A.T.M., D. N‐H., H.O. and M.C.B. synthesized NU‐FL and QX‐FL; M.L.B., N.L.T., A.A.L., S.A.M, K.T.M. and C.H.G. analysed the data; M.L.B., N.L.T., A.J.W., C.H.G., A.A.L., K.T.M., S.A.M. and M.C.B. wrote the paper.

## Conflict of interest

The authors declare no conflicts of interest.

## Declaration of transparency and scientific rigour

This Declaration acknowledges that this paper adheres to the principles for transparent reporting and scientific rigour of preclinical research recommended by funding agencies, publishers and other organisations engaged with supporting research.

## Supporting information


**Figure S1** Heterologous expression of functional Na_v_1.5 channels in HEK293 cells. (A) A profile of Coomassie stained proteins following SDS‐PAGE (left) and the corresponding immunoblot of Na_v_1.5 channels in H‐Flp/ Na_v_1.5 cells (right)(see Supplementary Methods). The Na_v_1.5‐null H‐Flp host cell line was used as control. (B) Combined immunofluorescence and brightfield images shows recombinant Na_v_1.5 channels at plasma membranes (red) in H‐Flp/ Na_v_1.5 cells and confirmed the absence of Na_v_1.5 protein in control H‐Flp cells. Nuclei were counterstained with DAPI (blue). Scale bar is 20 μm. (C) Voltage‐dependent activation of the Na^+^ current (I_Na_) in H‐Flp/ Na_v_1.5 cells was measured using 100 ms, 10 mV increment steps between −80 and +60 mV from a holding potential of −120 mV.
**Figure S2** Use‐dependent block (UDB) of voltage gated Na^+^ channel current (I_Na_) by flecainide analogues, NU‐FL and QX‐FL. I_Na_ traces prior to UDB (in the absence of flecainide (A) or during a 1 Hz protocol (B)) and at steady‐state UDB following the completion of a 10 Hz activation protocol in the presence of NU‐FL or QX‐FL. (C) Data for 10 Hz UDB, expressed as a fraction of non‐blocked, steady state I_Na_ is given as mean ± SEM (n = 8 (10 μM NU‐FL), n = 9 (100 μM NU‐FL and 10 μM QX‐FL).
**Figure S3** Flecainide has no effect on spontaneous Ca^2+^ release transients, through hRyR2 channels, in transfected HEK293 cells. (A) Sample traces showing spontaneous Ca^2+^ release events from three different cells expressing hRyR2 channels in the absence and in the presence of flecainide (5 μM). Flecainide had no effect on Ca^2+^ spike amplitude (B), duration (C), inter‐transient duration (D) or frequency (E) (n = 8). Supplementary Table. Ca^2+^ spark parameters.

Supporting info items

## References

[bph13521-bib-0001] Ahern CA , Eastwood AL , Dougherty DA , Horn R (2008). Electrostatic contributions of aromatic residues in the local anesthetic receptor of voltage‐gated sodium channels. Circ Res 102: 86–94. 17967784 10.1161/CIRCRESAHA.107.160663

[bph13521-bib-0002] Alexander SPH , Catterall WA , Kelly E , Marrion N , Peters JA , Benson HE *et al.* (2015a). The Concise Guide to PHARMACOLOGY 2015/16: Voltage‐gated ion channels. Br J Pharmacol 172: 5904–5941. 26650441 10.1111/bph.13349 PMC4718209

[bph13521-bib-0003] Alexander SPH , Peters JA , Kelly E , Marrion N , Benson HE , Faccenda E *et al.* (2015b). The Concise Guide to PHARMACOLOGY 2015/16: Ligand‐gated ion channels. Br J Pharmacol 172: 5870–5903. 26650440 10.1111/bph.13350 PMC4718212

[bph13521-bib-0004] Alexander SPH , Kelly E , Marrion N , Peters JA , Benson HE , Faccenda E *et al.* (2015c). The Concise Guide to PHARMACOLOGY 2015/16: Transporters. Br J Pharmacol 172: 6110–6202. 26650446 10.1111/bph.13355 PMC4718215

[bph13521-bib-0005] Bannister ML , Thomas NL , Sikkel MB , Mukherjee S , Maxwell C , MacLeod KT *et al.* (2015). The mechanism of flecainide action in CPVT does not involve a direct effect on RyR2. Circ Res 116: 1324–1335. 25648700 10.1161/CIRCRESAHA.116.305347

[bph13521-bib-0006] Curtis MJ , Bond RA , Spina D , Ahluwalia A , Alexander SPA , Giembycz MA *et al.* (2015). Experimental design and analysis and their reporting: new guidance for publication in BJP. Br J Pharmacol 172: 3461–3471. 26114403 10.1111/bph.12856 PMC4507152

[bph13521-bib-0007] Follmer CH , Colatsky TJ (1990). Block of delayed rectifier potassium current, IK, by flecainide and E‐4031 in cat ventricular myocytes. Circulation 82: 289–293. 2114236 10.1161/01.cir.82.1.289

[bph13521-bib-0008] Galimberti ES , Knollmann BC (2011). Efficacy and potency of class I antiarrhythmic drugs for suppression of Ca^2^ ^+^ waves in permeabilized myocytes lacking calsequestrin. J Mol Cell Cardiol 51: 760–768. 21798265 10.1016/j.yjmcc.2011.07.002 PMC3184367

[bph13521-bib-0009] Gillespie D , Fill M (2008). Intracellular calcium release channels mediate their own countercurrent: the ryanodine receptor case study. Biophys J 95: 3706–3714. 18621826 10.1529/biophysj.108.131987 PMC2553138

[bph13521-bib-0010] Hilliard FA , Steele DS , Laver D , Yang Z , Le Marchand SJ , Chopra N *et al.* (2010). Flecainide inhibits arrhythmogenic Ca^2^ ^+^ waves by open state block of ryanodine receptor Ca^2^ ^+^ release channels and reduction of Ca^2^ ^+^ spark mass. J Mol Cell Cardiol 48: 293–301. 19835880 10.1016/j.yjmcc.2009.10.005 PMC2813417

[bph13521-bib-0011] Hwang HS , Hasdemir C , Laver D , Mehra D , Turhan K , Faggioni M *et al.* (2011). Inhibition of cardiac Ca^2^ ^+^ release channels (RyR2) determines efficacy of class I antiarrhythmic drugs in catecholaminergic polymorphic ventricular tachycardia. Circ Arrhythm Electrophysiol 4: 128–135. 21270101 10.1161/CIRCEP.110.959916 PMC3667204

[bph13521-bib-0012] Kilkenny C , Browne WJ , Cuthill IC , Emerson M , Altman DG (2010). Improving bioscience research reporting: the ARRIVE guidelines for reporting animal research. PLoS Biol 8: e1000412. 20613859 10.1371/journal.pbio.1000412 PMC2893951

[bph13521-bib-0013] Lee YS , Maruyama M , Chang PC , Park HW , Rhee KS , Hsieh YC *et al.* (2012). Ryanodine receptor inhibition potentiates the activity of Na channel blockers against spontaneous calcium elevations and delayed afterdepolarizations in Langendorff‐perfused rabbit ventricles. Heart Rhythm 9: 1125–1132. 22387372 10.1016/j.hrthm.2012.02.031 PMC3572724

[bph13521-bib-0014] Liu H , Atkins J , Kass RS (2003). Common molecular determinants of flecainide and lidocaine block of heart Na^+^ channels: evidence from experiments with neutral and quaternary flecainide analogues. J Gen Physiol 121: 199–214. 12601084 10.1085/jgp.20028723 PMC2217334

[bph13521-bib-0015] Liu N , Denegri M , Ruan Y , Avelino‐Cruz JE , Perissi A , Negri S *et al.* (2011). Short communication: flecainide exerts an antiarrhythmic effect in a mouse model of catecholaminergic polymorphic ventricular tachycardia by increasing the threshold for triggered activity. Circ Res 109: 291–295. 21680895 10.1161/CIRCRESAHA.111.247338

[bph13521-bib-0016] Liu H , Tateyama M , Clancy CE , Abriel H , Kass RS (2002). Channel openings are necessary but not sufficient for use‐dependent block of cardiac Na^+^ channels by flecainide: evidence from the analysis of disease‐linked mutations. J Gen Physiol 120: 39–51. 12084774 10.1085/jgp.20028558 PMC2311398

[bph13521-bib-0017] Mackrill JJ (2010). Ryanodine receptor calcium channels and their partners as drug targets. Biochem Pharmacol 79: 1535–1543. 20097179 10.1016/j.bcp.2010.01.014

[bph13521-bib-0018] Mason SA , Viero C , Euden J , Bannister M , West D , Chen SR *et al.* (2012). The contribution of hydrophobic residues in the pore‐forming region of the ryanodine receptor channel to block by large tetraalkylammonium cations and Shaker B inactivation peptides. J Gen Physiol 140: 325–339. 22930804 10.1085/jgp.201210851 PMC3434103

[bph13521-bib-0019] McGarry SJ , Williams AJ (1994). Activation of the sheep cardiac sarcoplasmic reticulum Ca^2^ ^+^‐release channel by analogues of sulmazole. Br J Pharmacol 111: 1212–1220. 8032608 10.1111/j.1476-5381.1994.tb14874.x PMC1910124

[bph13521-bib-0020] McGrath JC , Lilley E (2015). Implementing guidelines on reporting research using animals (ARRIVE etc.): new requirements for publication in BJP. Br J Pharmacol 172: 3189–3193. 25964986 10.1111/bph.12955 PMC4500358

[bph13521-bib-0021] Mead FC , Williams AJ (2004). Electrostatic mechanisms underlie neomycin block of the cardiac ryanodine receptor channel (RyR2). Biophys J 87: 3814–3825. 15361409 10.1529/biophysj.104.049338 PMC1304893

[bph13521-bib-0022] Mehra D , Imtiaz MS , van Helden DF , Knollmann BC , Laver DR (2014). Multiple modes of ryanodine receptor 2 inhibition by flecainide. Mol Pharmacol 86: 696–706. 25274603 10.1124/mol.114.094623 PMC4244595

[bph13521-bib-0023] Melgari D , Zhang Y , El Harchi A , Dempsey CE , Hancox JC (2015). Molecular basis of hERG potassium channel blockade by the class Ic antiarrhythmic flecainide. J Mol Cell Cardiol 86: 42–53. 26159617 10.1016/j.yjmcc.2015.06.021 PMC4564290

[bph13521-bib-0024] Mukherjee S , Thomas NL , Williams AJ (2012). A mechanistic description of gating of the human cardiac ryanodine receptor in a regulated minimal environment. J Gen Physiol 140: 139–158. 22802361 10.1085/jgp.201110706 PMC3409104

[bph13521-bib-0025] Napolitano C (2016). Flecainide monotherapy for catecholaminergic polymorphic ventricular tachycardia: perspectives and limitations. Heart Rhythm 13: 614–615. 26498259 10.1016/j.hrthm.2015.10.030

[bph13521-bib-0026] Padfield GJ , AlAhmari L , Lieve KV , AlAhmari T , Roston TM , Wilde AA *et al.* (2016). Flecainide monotherapy is an option for selected patients with catecholaminergic polymorphic ventricular tachycardia intolerant of β‐blockade. Heart Rhythm 13: 609–613. 26416620 10.1016/j.hrthm.2015.09.027

[bph13521-bib-0027] Paul AA , Witchel HJ , Hancox JC (2002). Inhibition of the current of heterologously expressed HERG potassium channels by flecainide and comparison with quinidine, propafenone and lignocaine. Br J Pharmacol 136: 717–729. 12086981 10.1038/sj.bjp.0704784 PMC1573407

[bph13521-bib-0029] Qin F , Li L (2004). Model‐based fitting of single‐channel dwell‐time distributions. Biophys J 87: 1657–1671. 15345545 10.1529/biophysj.103.037531 PMC1304571

[bph13521-bib-0030] Ragsdale DS , McPhee JC , Scheuer T , Catterall WA (1994). Molecular determinants of state‐dependent block of Na^+^ channels by local anesthetics. Science 265: 1724–1728. 8085162 10.1126/science.8085162

[bph13521-bib-0031] Ragsdale DS , McPhee JC , Scheuer T , Catterall WA (1996). Common molecular determinants of local anesthetic, antiarrhythmic, and anticonvulsant block of voltage‐gated Na^+^ channels. Proc Natl Acad Sci 93: 9270–9275. 8799190 10.1073/pnas.93.17.9270 PMC38631

[bph13521-bib-0032] Santulli G , Marks AR (2015). Essential roles of intracellular calcium release channels in muscle, brain, metabolism, and aging. Curr Mol Pharmacol 8: 206–222. 25966694 10.2174/1874467208666150507105105

[bph13521-bib-0033] Sato M , O'Gara P , Harding SE , Fuller SJ (2005). Enhancement of adenoviral gene transfer to adult rat cardiomyocytes *in vivo* by immobilization and ultrasound treatment of the heart. Gene Ther 12: 936–941. 15759019 10.1038/sj.gt.3302476

[bph13521-bib-0034] Savio‐Galimberti E , Knollmann BC (2015). Channel activity of cardiac ryanodine receptors (RyR2) determines potency and efficacy of flecainide and R‐propafenone against arrhythmogenic calcium waves in ventricular cardiomyocytes. PLoS One 10: e0131179. 26121139 10.1371/journal.pone.0131179 PMC4488248

[bph13521-bib-0035] Sikkel MB , Collins TP , Rowlands C , Shah M , O'Gara P , Williams AJ *et al.* (2013a). Flecainide reduces Ca^2^ ^+^ spark and wave frequency via inhibition of the sarcolemmal sodium current. Cardiovasc Res 98: 286–296. 23334259 10.1093/cvr/cvt012 PMC3714924

[bph13521-bib-0036] Sikkel MB , Collins TP , Rowlands C , Shah M , O'Gara P , Williams AJ *et al.* (2013b). Triple mode of action of flecainide in catecholaminergic polymorphic ventricular tachycardia: reply. Cardiovasc Res 98: 327–328. 23536607 10.1093/cvr/cvt068

[bph13521-bib-0037] Sitsapesan R , Williams AJ (1994). Gating of the native and purified cardiac SR Ca^2^ ^+^‐release channel with monovalent cations as permeant species. Biophys J 67: 1484–1494. 7819484 10.1016/S0006-3495(94)80622-8 PMC1225511

[bph13521-bib-0038] Southan C , Sharman JL , Benson HE , Faccenda E , Pawson AJ , Alexander SPH *et al.* (2016). The IUPHAR/BPS Guide to PHARMACOLOGY in 2016: towards curated quantitative interactions between 1300 protein targets and 6000 ligands. Nucl Acids Res 44 (Database Issue): D1054–D1068. 26464438 10.1093/nar/gkv1037 PMC4702778

[bph13521-bib-0039] Steele DS , Hwang HS , Knollmann BC (2013). Triple mode of action of flecainide in catecholaminergic polymorphic ventricular tachycardia. Cardiovasc Res 98: 326–327. 23512981 10.1093/cvr/cvt059 PMC3657388

[bph13521-bib-0040] Tinker A , Williams AJ (1993). Charged local anesthetics block ionic conduction in the sheep cardiac sarcoplasmic reticulum calcium release channel. Biophys J 65: 852–864. 8218909 10.1016/S0006-3495(93)81104-4 PMC1225786

[bph13521-bib-0041] Tinker A , Lindsay AR , Williams AJ (1992a). Large tetraalkyl ammonium cations produce a reduced conductance state in the sheep cardiac sarcoplasmic reticulum Ca^2^ ^+^‐release channel. Biophys J 61: 1122–1132. 1318091 10.1016/S0006-3495(92)81922-7 PMC1260377

[bph13521-bib-0042] Tinker A , Lindsay AR , Williams AJ (1992b). Block of the sheep cardiac sarcoplasmic reticulum Ca^2^ ^+^‐release channel by tetra‐alkyl ammonium cations. J Membr Biol 127: 149–159. 1625325 10.1007/BF00233287

[bph13521-bib-0043] van der Werf C , Kannankeril PJ , Sacher F , Krahn AD , Viskin S , Leenhardt A *et al.* (2011). Flecainide therapy reduces exercise‐induced ventricular arrhythmias in patients with catecholaminergic polymorphic ventricular tachycardia. J Am Coll Cardiol 57: 2244–2254. 21616285 10.1016/j.jacc.2011.01.026 PMC3495585

[bph13521-bib-0044] van der Werf C , Nederend I , Hofman N , van Geloven N , Ebink C , Frohn‐Mulder IM *et al.* (2012). Familial evaluation in catecholaminergic polymorphic ventricular tachycardia: disease penetrance and expression in cardiac ryanodine receptor mutation carrying relatives. Circ Arrhythm Electrophysiol 5: 748–756. 22787013 10.1161/CIRCEP.112.970517

[bph13521-bib-0045] Watanabe H , Chopra N , Laver D , Hwang HS , Davies SS , Roach DE *et al.* (2009). Flecainide prevents catecholaminergic polymorphic ventricular tachycardia in mice and humans. Nat Med 15: 380–383. 19330009 10.1038/nm.1942 PMC2904954

[bph13521-bib-0046] Watanabe H , van der Werf C , Roses‐Noguer F , Adler A , Sumitomo N , Veltmann C *et al.* (2013). Effects of flecainide on exercise‐induced ventricular arrhythmias and recurrences in genotype‐negative patients with catecholaminergic polymorphic ventricular tachycardia. Heart Rhythm 10: 542–547. 23286974 10.1016/j.hrthm.2012.12.035 PMC3809106

