# Supplementary material for: Effect of flecainide derivatives on sarcoplasmic reticulum calcium release suggests a lack of direct action on the cardiac ryanodine receptor
Source: Br J Pharmacol. 2016 Jun 29;173(15):2446–59. doi: 10.1111/bph.13521 (PMC4945764; doi:10.1111/bph.13521)
Supplement: Supplementary file 1 — Supporting info items [file BPH-173-2446-s001.pdf]

**Supplementary Material**

# Effect of flecainide derivatives on sarcoplasmic reticulum calcium release suggests a lack of direct action on the cardiac ryanodine receptor

**Running title: Flecainide derivatives and ryanodine receptors**

**Mark L. Bannister[1], Anita Alvarez-Laviada[2], N. Lowri Thomas[1], Sammy A. Mason[1], Sharon Coleman[1], Christo L. du Plessis[3], Abbygail T. Moran[3], David Neill-Hall[3], Hasnah Osman[4], Mark C. Bagley[3], Kenneth T. MacLeod[2], Christopher H. George[*1], Alan J. Williams[*1]**

[*]Correspondence
Christopher H. George georgech@cardiff.ac.uk; Alan J.Williams williamsaj9@cardiff.ac.uk

[1]Wales Heart Research Institute, Cardiff University School of Medicine, Cardiff, CF14 4XN, UK
[2]Myocardial Function Section, National Heart and Lung Institute, Imperial College London, London, UK
[3]Department of Chemistry, School of Life Sciences, University of Sussex, Falmer, Brighton, East Sussex, UK
[4]School of Chemical Sciences, Universiti Sains Malaysia, 11800, Penang, Malaysia

**Supplementary Methods**

***Synthesis of reagents for NU-FL production***

*Methyl 2,5-di(2,2,2-trifluoroethoxy)benzoate.* A solution of methyl 2,5-dihydroxybenzoate (Aldrich) (3.02 g, 18 mmol) in acetone (30 mL) was added dropwise to a mixture of $K_2CO_3$ (7.28 g, 53 mmol) and 2,2,2-trifluoroethyl perfluorobutylsulfonate (10.5 mL, 45 mmol) in acetone (30 mL). The reaction mixture was stirred and heated under reflux for 31 h, filtered hot and evaporated *in vacuo*. Purification by flash chromatography on silica, eluting with light petrolium–$Et_2O$ (1:1) gave the *title compound* (5.41 g, 90%) as a colourless solid, mp 40.0–42.0 ℃ (found: 355.0362. $C_{12}H_{10}F_6NaO_4$ [*M*] requires 355.0375) (purity > 98% by LCMS, gradient eluting with acidified MeCN–$H_2O$, 30-90%, $t_R$ 19.8 min) ($R_f$ 0.48 on $SiO_2$ eluting with light petroleum-$Et_2O$, 1:1); IR $\nu_{max}$ 2964 (CH), 1701 (C=O), 1501, 1446 1265; $^1$H NMR (500 MHz; $CD_3OD$) $\delta$ 7.41 (1H, d, *J* 3, 6-H), 7.22 (1H, dd, *J* 3,9, 4-H), 7.17 (1H, d, *J* 9, 3-H), 4.55 (2H, q, *J* 8.5, $CH_2$), 4.53 (2H, q, *J* 8.5, $CH_2$), 3.89 (3H, s, Me); $^{13}$C NMR (125 MHz, $CD_3OD$) 165.8 (C=O), 152.7 (C), 152.0 (C), 123.64 (q, $^1J_{C-F}$ 276, $CF_3$) 123.62 (q, $^1J_{C-F}$ 276, $CF_3$), 122.9 (C), 120.1 (CH), 118.2 (CH), 117.1 (CH), 67.9 (q, $^2J_{C-F}$ 35, $CH_2$), 65.9 (q, $^2J_{C-F}$ 35, $CH_2$), 52.3 (Me); MS (EI) *m/z* (rel. intensity) 332 ($M^{\bullet+}$, 100%), 301 (84), 249 (46), 231 (38).

*2,5-(2,2,2-Trifluorethoxy)benzoic acid.* Methyl 2,5-(2,2,2-trifluoroethoxy)benzoate (4.8 g, 14 mmol) was added to a stirred solution of NaOH (0.58 g, 14 mmol) in $H_2O$ (60 mL). The reaction mixture was heated under reflux for 20 h, cooled and partitioned between $H_2O$ (50 mL) and $Et_2O$ (50 mL). The aqueous layer was separated, acidified with hydrochloric acid (1 M) giving a colourless precipitate, and extracted with EtOAc (5 × 30 mL). The organic extracts were combined, washed with brine (2 × 40 mL), dried ($MgSO_4$) and evaporated *in vacuo* to give the *title compound* (4.09 g, 92%) as a colourless solid, mp 116.9–117.9 °C (found: 341.0207. $C_{11}H_8F_6NaO_4$ [*M*] requires 341.0219) (purity > 98% by LCMS, gradient eluting with acidified MeCN–$H_2O$, 30-90%, $t_R$ 16.6 min) ($R_f$ 0.58 on $SiO_2$ eluting with light petroleum–EtOAc, 1:3); IR $\nu_{max}$ 3000 (br, OH), 1678 (C=O), 1282, 1156; $^1$H NMR (500 MHz; $CDCl_3$) $\delta$ 7.65 (1H, d, *J* 3, 6-H), 7.22 (1H, dd, *J* 3,9, 4-H), 7.07 (1H, d, *J* 9, 3-H), 4.50 (2H, q, *J* 8, $CH_2$) 4.40 (2H, q, *J* 8, $CH_2$); $^{13}$C NMR (125 MHz; $CDCl_3$) 166.7 (C=O), 153.1 (C), 152.4 (C), 123.0 (q, $^1J_{C-F}$ 277, $CF_3$), 122.9 (q, $^1J_{C-F}$ 277, $CF_3$), 122.7 (C), 120.8 (CH), 118.0 (CH), 117.8 (CH), 68.5 (q, $^2J_{C-F}$ 36, $CH_2$), 66.5 (q, $^2J_{C-F}$ 36, $CH_2$); MS (EI) *m/z* (rel. intensity) 318 ($M^{\bullet+}$, 100%), 235 (50), 169 (26), 157 (32), 115 (37).

*N-[2-(4-Morpholinyl)ethyl]-2,5-bis(2,2,2-trifluoroethoxy)benzamide (NU-FL).* Oxalyl chloride (2.8 mL, 33 mmol), was added dropwise to a solution of 2,5-(2,2,2-trifluoroethoxy)benzoic acid (3.28 g, 10 mmol) in dichloromethane (150 mL). The reaction mixture was stirred under nitrogen at room temperature for 21 h, diluted with dichloromethane (100 mL) and washed sequentially with saturated aqueous sodium hydrogen carbonate solution (2 × 40 mL) and brine (3 × 30 mL). The organic layer was dried ($MgSO_4$) and evaporated *in vacuo* to give the crude intermediate, 2,5-bis(2,2,2-trifluoroethoxy)benzoyl chloride (3.20 g). 2,5-Bis(2,2,2-trifluoroethoxy)benzoyl chloride (3.20 g, 9.5 mmol) was dissolved in THF (150 mL) and added dropwise to a mixture of 4-(2-aminoethyl)morpholine (1.24 mL, 9.5 mmol) and triethylamine (2.65 mL, 19 mmol). The reaction mixture was stirred under nitrogen at room temperature for 19 h, evaporated *in vacuo* and then partitioned between EtOAc and saturated aqueous sodium hydrogen carbonate solution (30 mL). The organic layer was washed with saturated aqueous sodium hydrogen carbonate solution (2 × 30 mL), dried ($MgSO_4$) and evaporated *in vacuo*. Purification by flash chromatography on silica, eluting with light petrolium–EtOAc (1:1), gave the *title compound* (2.23 g, 52%) as a

colourless solid, mp 80.3–81.1 °C (found: 431.1395. $C_{17}H_{21}F_6N_2O_4$ [*M*] requires 431.1400) (purity > 98% by LCMS, gradient eluting with acidified MeCN–$H_2O$, 5-95%, $t_R$ 11.7 min) ($R_f$ 0.25 on $SiO_2$ eluting with light petroleum–EtOAc, 1:3); IR $\nu_{max}$ 3284 (C=O), 2863 (CH), 1643 (C=O). 1284, 1223, 1156; $^1$H NMR (500 MHz; $CDCl_3$) $\delta$ 7.76 (1H, d, *J* 3, 6-H), 7.75 (1H, s, exch $D_2O$, NH) 7.11 (1H, dd, *J* 3,9, 4-H), 6.93 (1H, d, *J* 9, 3-H), 4.47 (2H, q, *J* 8, 1′-$CH_2$), 4.39 (2H, q, *J* 8, 1′-$CH_2$), 3.72 (4H, t, *J* 5, 2‴,6‴-H), 3.61 (2H, q, *J* 6, 1″-H), 2.59 (2H, t, *J* 6, 2″-H), 2.49 (4H, t, *J* 5, 3‴,5‴-H); $^{13}$C NMR (125 MHz; $CD_3OD$) 165.7 (C=O), 152.8 (C), 150.2 (C), 123.7 (q, $^1J_{C\text{-}F}$ 276, $CF_3$), 123.6 (q, $^1J_{C\text{-}F}$ 276, $CF_3$), 124.9 (C), 118.9 (CH), 116.6 (CH), 115.6 (CH), 66.7 (q, $^2J_{C\text{-}F}$ 35, $CH_2$), 66.3 ($CH_2$), 65.8 (q, $^2J_{C\text{-}F}$ 35, $CH_2$), 57.0 ($CH_2$), 53.3 ($CH_2$), 36.3 ($CH_2$); MS (EI) *m/z* (rel. intensity) 430 ($M^{\bullet+}$, 29%), 387 (18), 113 (23), 100 (100).

***Stable expression of Na$_v$1.5 in human embryonic kidney (HEK) cells.***
cDNA corresponding to the open reading frame (ORF) of the human *scn5A* gene (Entrez gene ID:6331) was synthesised and cloned into pUC57 (GenScript). The ORF minus the stop codon (-tga) was amplified as a single PCR product and sub-cloned into pcDNA5-FRT-V5-His-TOPO (Invitrogen). The resulting plasmid (pcDNA5-FRT-scn5A-V5-His) encoded $Na_v$1.5 (NP_932173; 2016 amino acids) fused to V5 and 6xHis elements via an 11-mer linker (KGELGTELGSE). DNA sequences were verified using BigDye Terminator v3.1 cycle sequencing and a Prism 3700 analyser (Applied Biosystems). HEK cells containing a stably integrated flippase recombination target (FRT) (HEK-Flp; Invitrogen) were maintained in Dulbecco's modified Eagle Medium (DMEM) containing 4.5 g/L glucose and supplemented with glutamine (2 mM), penicillin/streptomycin (1 unit/ml and 1 µg/ml, respectively) and zeocin (100 µg/ml). HEK-Flp were co-transfected with pcDNA5-FRT-scn5A-V5-His and pOG44, a plasmid encoding *flp* recombinase (Invitrogen), using Lipofectamine as described (George *et al.*, 2003). HEK-Flp-scn5A selectants (referred to subsequently as H-Flp/$Na_v$1.5) were maintained in supplemented DMEM containing zeocin (100 µg/ml) and hygromycin B (300 µg/ml). HEK-Flp (H-Flp) were used as $Na_v$1.5-null controls for immunofluorescence and western blotting.

***Immunofluorescence and western blotting detection of recombinant Na$_v$1.5***
For immunofluorescence, cells were plated on poly-L-lysine-coated coverslips and after 24h cells were fixed in ice-cold methanol (10 min), washed with phosphate buffered saline (PBS containing (in mM): NaCl (140), KCl (2.7), $Na_2HPO_4$ (10), $NaH_2PO_4$ (2), pH7.4)) and permeabilised with Triton (0.2% (v/v)) in PBS for 10 min. Following washing with PBS non-specific antibody binding was blocked using 5% (v/v) foetal bovine serum (FBS) in PBS. Cells were incubated with a mouse monoclonal anti-V5 antibody (R960-25; Invitrogen) diluted 1:1000 in filtered PBS overnight at 4°C. Following washing with PBS, cells were incubated with donkey anti-mouse Alexa-546 conjugated secondary antibody (Invitrogen). $Na_v$1.5 immunolocalisation was visualised using confocal microscopy as described (Lewis *et al.*, 2015).
For western blotting, microsomal fractions were prepared as described (Mukherjee *et al.*, 2012) and were separated using 10% (v/v) acrylamide SDS-PAGE on a large Hoefer system run for >15 h at 40 mA and at 4°C[1]. Proteins were transferred to methanol-treated, rehydrated PVDF membranes using semi-dry apparatus (GE Healthcare) at 60 mA for 2 h. Membranes were washed with Tween 20-containing Tris-buffered saline (TBS-T), blocked for 1 h in TBS-T containing 5 % (w/v) non-fat powdered milk and incubated for approximately 15 h at 4 °C in PBS containing FBS (2% (v/v)) and a 1:100 dilution of rabbit polyclonal anti-$Na_v$1.5 antibody raised against a C-terminal epitope (1978-2016 amino acids) (ASC-013; Alomone Labs). Membranes were then washed with TBS-T and incubated with a goat-anti-rabbit HRP-

conjugated antibody (1:10,000 dilution) (Sigma) in PBS containing FBS (2% (v/v)). Membranes were washed with TBS-T and HRP enzymic activity was detected using chemiluminescence (ECL, GE Healthcare) and developed to film (HyperfilmECL, GE Healthcare).

***Measurement of voltage gated sodium current***
H-Flp/$Na_v$1.5 were used to assess UDB by QX-FL and NU-FL. Cells were prepared at a density of 0.4 x $10^6$ in 35 mm polystyrene dishes (Scientific Lab Supplies, Nottingham, UK) and were used within 48-72 hours of seeding. Cells were continuously superfused at 21 °C in an extracellular solution containing (mM) 20 NaCl, 140 NMDG, 5 KCl, 1 $MgCl_2$, 2 $CaCl_2$, 5 HEPES, 10 glucose (pH 7.4, HCl). Whole cell sodium current ($I_{Na}$) was measured using patch electrodes with a resistance of 2-3 MΩ containing an intracellular solution of (mM) 120 CsF, 20 $CsCl_2$, 5 EGTA, 5 HEPES (pH 7.2, CsOH). QX-FL and NU-FL were prepared as 10 mM stocks in DMSO and were diluted in intracellular and extracellular solution at 10 µM and 10/100 µM respectively. UDB of $I_{Na}$ was achieved by the application of a 600 pulse, 10 Hz conditioning train (pre-sweeps) of voltage clamp steps from a holding potential of -110 mV to a 25 ms test potential of -10 mV (Liu *et al.*, 2003). Following a sequence of 10 conditioning pulses, $I_{Na}$ was recorded with a single step (total of 60 sweeps) using the same voltage step protocol (at 1 Hz) permitting the use of post-hoc, P/4 leak subtraction. Unless otherwise stated, UDB (%) was calculated as the % difference between $I_{Na}$ at the $1^{st}$ and $60^{th}$ sweep pulse. Data were acquired at 100 kHz and low pass filtered using a 10 kHz Bessel filter. To assess UDB with extracellular NU-FL, $I_{Na}$ was compared at steady state prior to and following application of NU-FL to the ECS and expressed as UDB (%). To examine UDB with intracellular QX-FL, a 1 Hz series of voltage steps was applied (-110 mV to -10 mV for 150 ms) to the cells, following which, $I_{Na}$ was compared during the 10 Hz UDB protocol and expressed as UDB (%).

***Imaging of spontaneous $Ca^{2+}$ release events in HEK293 cells expressing hRyR2***
HEK293 cells (~1 x $10^5$) were seeded on poly-D-Lysine coated glass cover chambers (MatTek) before Effectene® (Qiagen) mediated transfection using pcDNA3/eGFP-hRyR2 (~0.1µg/cover chamber). Cells were loaded with fluo-3 AM (10 µM, LifeTech) 48 hours post-transfection before immersing in Krebs-Ringer Hepes (KRH) solution (containing (mM), 120 NaCl, 5.5 glucose, 25 HEPES, 4.8 KCl, 1.2 $KH_2PO_4$ and 1.3 $CaCl_2$) immediately prior to imaging. Cells were maintained at 37°C during experiments and data were acquired using a confocal microscope (SP5, Leica Microsystems) fitted with a 63x oil immersion objective. Recordings were carried out for 3 minutes in the absence of drug and for a further 3 minutes following the addition of a bolus of flecainide (5 µM). RyR2-mediated global $Ca^{2+}$ release events were acquired at a 512 x 512 pixel resolution at 5 frames/second.

**Supplementary Results**
In order to confirm the molecular properties of our NU-FL and QX-FL we examined the potential for UDB of voltage gated sodium current in HEK293 cells over-expressing recombinant $Na_v$1.5 using methodologies similar to those used by Liu and colleagues (Liu *et al.*, 2003). H-Flp/Nav1.5 cells were characterised by high-level expression of $Na_v$1.5 protein (Supplementary Figure 1A) that was homogeneously distributed at the plasma membrane (Supplementary Figure 1B). Voltage-elicited $I_{Na}$ currents were characteristic of $Na_v$1.5-mediated currents measured in native tissue (Colatsky, 1980; Brown *et al.*, 1981; Makielski *et al.*, 1987) and recombinant systems (Cribbs *et al.*, 1990; Gellens *et al.*, 1992; Chahine *et al.*, 1996; Wang *et al.*, 1996) (Supplementary Figure 1C). Following application of a 10 Hz,

600 pulse train of depolarizing steps (-110 mV to -10 mV) UDB was observed in the presence of either 10/100 µM NU-FL or 10 µM QX-FL when added to the extracellular or intracellular solutions respectively. In order to quantitively assess our NU-FL and QX-FL compounds we have compared the fractional block produced by these compounds at steady state (see Supplementary Figure 2). Similarly to previous findings (Liu *et al.*, 2003), we get comparable UDB at 10 and 100 µM NU-FL (NU-FL 10 µM, 19 ± 2%; 100 µM, 61 ± 1%, n = 9-10), whilst also seeing a greater affinity of QX-FL than NU-FL at 10 µM (QX-FL 10 µM, 69 ± 3% vs NU-FL 10 µM, 19 ± 2%, n = 9-10). The higher affinity of QX-FL in our hands (approximately 30% higher UDB) may be explained by methodological differences, particularly the definition of 'steady state' in the study by Liu et al (Liu *et al.*, 2003), but nevertheless, QX-FL in our hands exhibits significant UDB of $I_{Na}$ when in the ICS.

Flecainide (5 µM) had no effect on spontaneous $Ca^{2+}$ oscillations in HEK293 cells over-expressing recombinant human RyR2 (Thomas *et al.*, 2004) (Supplementary Figure 3). These cells are inherently devoid of $Na_v1.5$ (Supplementary Figure 1A) and these data corroborate the lack of effect of the drug on intracellular $Ca^{2+}$ release mediated exclusively via RyR2.

**Supplementary Table. $Ca^{2+}$ spark parameters**

| | Control-FL | FL | Control-QXFL | QXFL | Control-NUFL | NUFL |
|---|---|---|---|---|---|---|
| Cells / Hearts | 15 / 11 | 20 / 10 | 17 / 11 | 15 / 10 | 8 / 5 | 10 / 5 |
| Sparks | 1322 | 1034 | 1382 | 1051 | 441 | 627 |
| Frequency (sparks/100 µm/s) | 1.45±0.14 | 0.94±0.10* | 1.58±0.20 | 1.13±0.16* | 1.32±0.22 | 1.16±0.19 |
| Amplitude ($\Delta F/F_0$) | 0.20±0.01 | 0.19±0.01 | 0.23±0.02 | 0.20±0.01 | 0.20±0.01 | 0.20±0.01 |
| FWHM (µm) | 4.87±0.18 | 5.09±0.20 | 4.67±0.17 | 5.14±0.26 | 4.87±0.28 | 5.05±0.34 |
| FDHM (ms) | 104.78±10.25 | 104.25±6.08 | 86.98±7.46 | 115.91±11.67* | 128.36±19.53 | 113.52±15.42 |
| Time-to-peak (ms) | 59.70±6.59 | 58.34±3.97 | 49.74±3.68 | 64.63±5.83 | 79.15±13.69 | 67.39±8.36 |
| Max $Ca^{2+}$ upstroke ($\Delta F/F_0/dt$) | 8.43±0.48 | 7.61±0.37 | 9.95±1.13 | 8.06±0.45* | 7.76±0.45 | 7.74±0.33 |
| Tau (ms) | 360.18±95.53 | 277.80±41.01 | 167.27±35.42 | 350.69±85.81 | 323.04±67.73 | 257.81±57.97 |
| Mass ($\Delta F/F_0$ x 1.206 x $FWHM^3$) | 59.81±6.46 | 61.04±8.48 | 55.01±8.43 | 71.41±14.04 | 63.32±12.37 | 64.97±15.08 |

* p<0.05 compared with respective control group
All parameters across the three control groups were similar except for tau where p=0.0443 (ANOVA).

***References***

Brown AM, Lee KS, Powell T (1981). Sodium current in single rat heart muscle cells. J Physiol 318: 479-500.

Chahine M, Deschene I, Chen LQ, Kallen RG (1996). Electrophysiological characteristics of cloned skeletal and cardiac muscle sodium channels. Am J Physiol Heart Circ Physiol 271: H498-H506.

Colatsky TJ (1980). Voltage clamp measurements of sodium channel properties in rabbit cardiac Purkinje fibres. J Physiol 305: 215-34.

Cribbs LL, Satin J, Fozzard HA, Rogart RB (1990). Functional expression of the rat 1 $Na^+$ channel isoform: demonstration of properties characteristic of native cardiac $Na^+$ channels. FEBS Lett 275: 195-200.

Gellens ME, George Jr. AL, Chen L, Chahine M, Horn R, Barchi RL *et al.* (1992). Primary structure and functional expression of the human cardiac tetrodotoxin-insensitive voltage-dependent sodium channel. Proc Natl Acad Sci 89: 554-8.

George CH, Sorathia R, Bertrand BMA, Lai FA (2003). In situ modulation of the human cardiac ryanodine receptor (hRyR2) by FKBP12.6. Biochem J 370: 579-89.

Lewis KJ, Silvester NC, Barberini-Jammaers SR, Mason SA, Marsh SA, Lipka M *et al.* (2015). A new system for profiling drug-induced calcium signal perturbation in human embryonic stem cell-derived cardiomyocytes. J Biomol Screen 20: 330-40.

Liu H, Atkins J, Kass RS (2003). Common molecular determinants of flecainide and lidocainse block of heart $Na^+$ channels: evidence from experiments with neutral and quaternary flecainide analogues. J Gen Physiol 121: 199-214.

Makielski JC, Sheets MF, Hanck D, January CT, Fozzard HA (1987). Sodium current in voltage clamped internally perfused canine cardiac Purkinje cells. Biophys J 52: 1-11.

Mukherjee S, Thomas NL, Williams AJ (2012). A mechanistic description of gating of the human cardiac ryanodine receptor in a regulated minimal environment. J Gen Physiol 140: 139-58.

Thomas NL, George CH, Lai FA (2004). Functional heterogeneity of ryanodine receptor mutations associated with sudden cardiac death. Cardiovasc Res 64: 52-60.

Wang DW, Yazawa K, George Jr. AL, Bennett PB (1996). Characterization of human cardiac $Na^+$ channel mutations in the congenital long QT syndrome. Proc Natl Acad Sci 93: 13200-5.

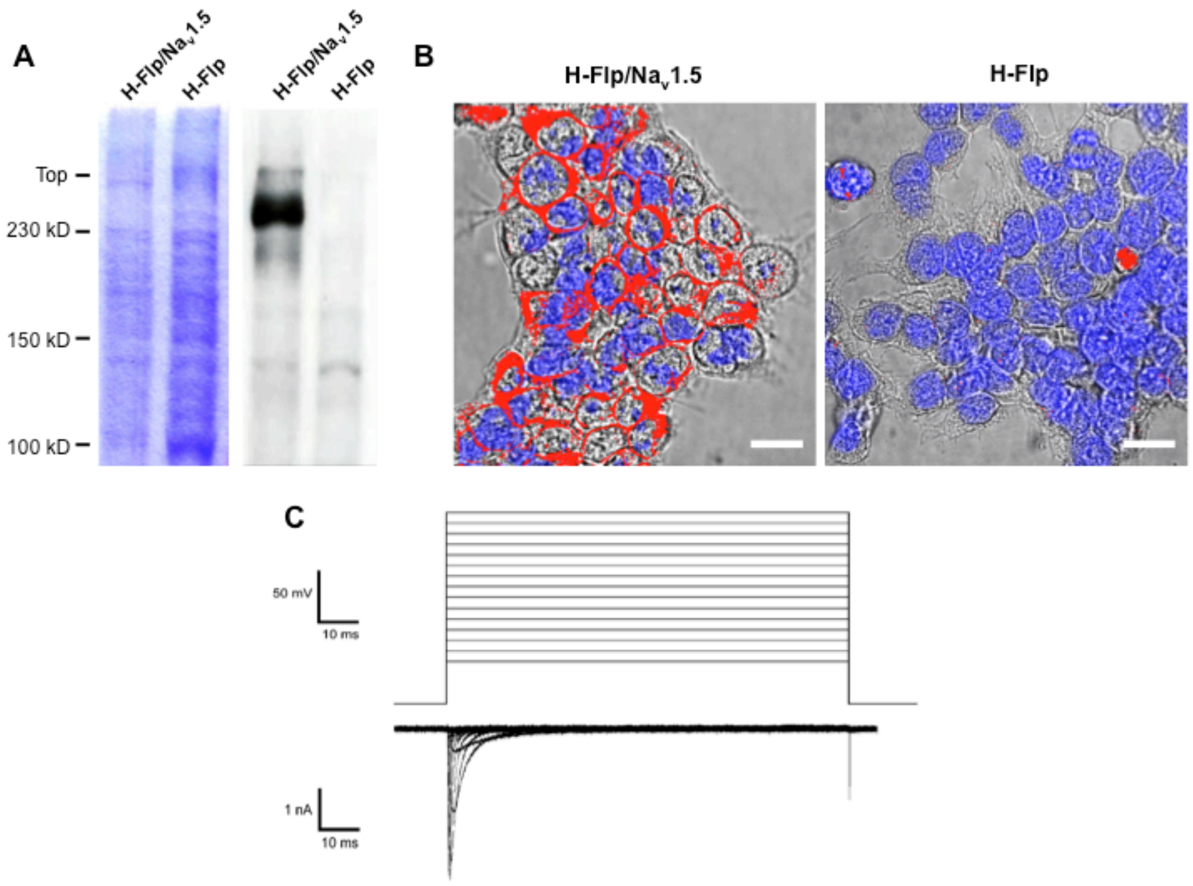


**Supplementary Figure 1.** Heterologous expression of functional $Na_v1.5$ in HEK293 cells. **(A)** A profile of Coomassie stained proteins following SDS-PAGE (left) and the corresponding immunoblot of $Na_v1.5$ expression in H-Flp/$Na_v1.5$ cells (right)(see Supplementary Methods). The $Na_v1.5$-null H-Flp host cell line was used as control. **(B)** Combined immunofluorescence and brightfield images shows recombinant $Na_v1.5$ at plasma membranes (red) in H-Flp/$Na_v1.5$ cells and confirmed the absence of $Na_v1.5$ protein in control H-Flp cells. Nuclei were counterstained with DAPI (blue). Scale bar is 20μm. **(C)** Voltage-dependent activation of $I_{Na}$ in H-Flp/$Na_v1.5$ cells was measured using 100ms, 10mV increment steps between -80 and +60mV from a holding potential of -120mV.

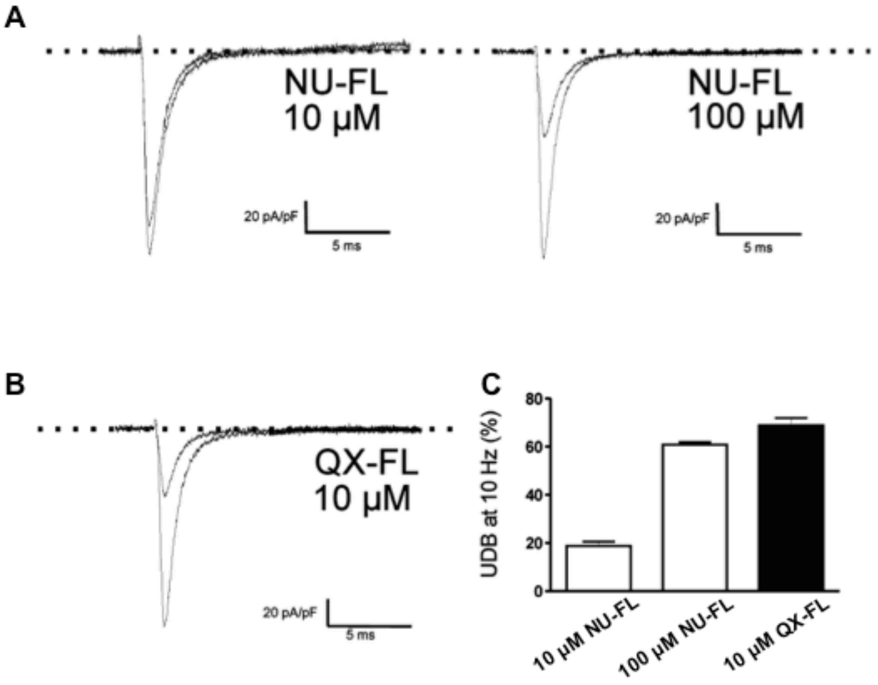


**Supplementary Figure 2.** Use-dependent block (UDB) of voltage gated sodium channel current ($I_{Na}$) by flecainide analogues, NU-FL and QX-FL. $I_{Na}$ traces prior to UDB (in the absence of flecainide **(A)** or during a 1 Hz protocol **(B)**) and at steady-state UDB following the completion of a 10 Hz activation protocol in the presence of NU-FL or QX-FL. **(C)** Data for 10 Hz UDB, expressed as a fraction of non-blocked steady state $I_{Na}$ is given as mean ± SEM (n = 8 (10 μM NU-FL), n=9 (100 μM NU-FL and 10 μM QX-FL).

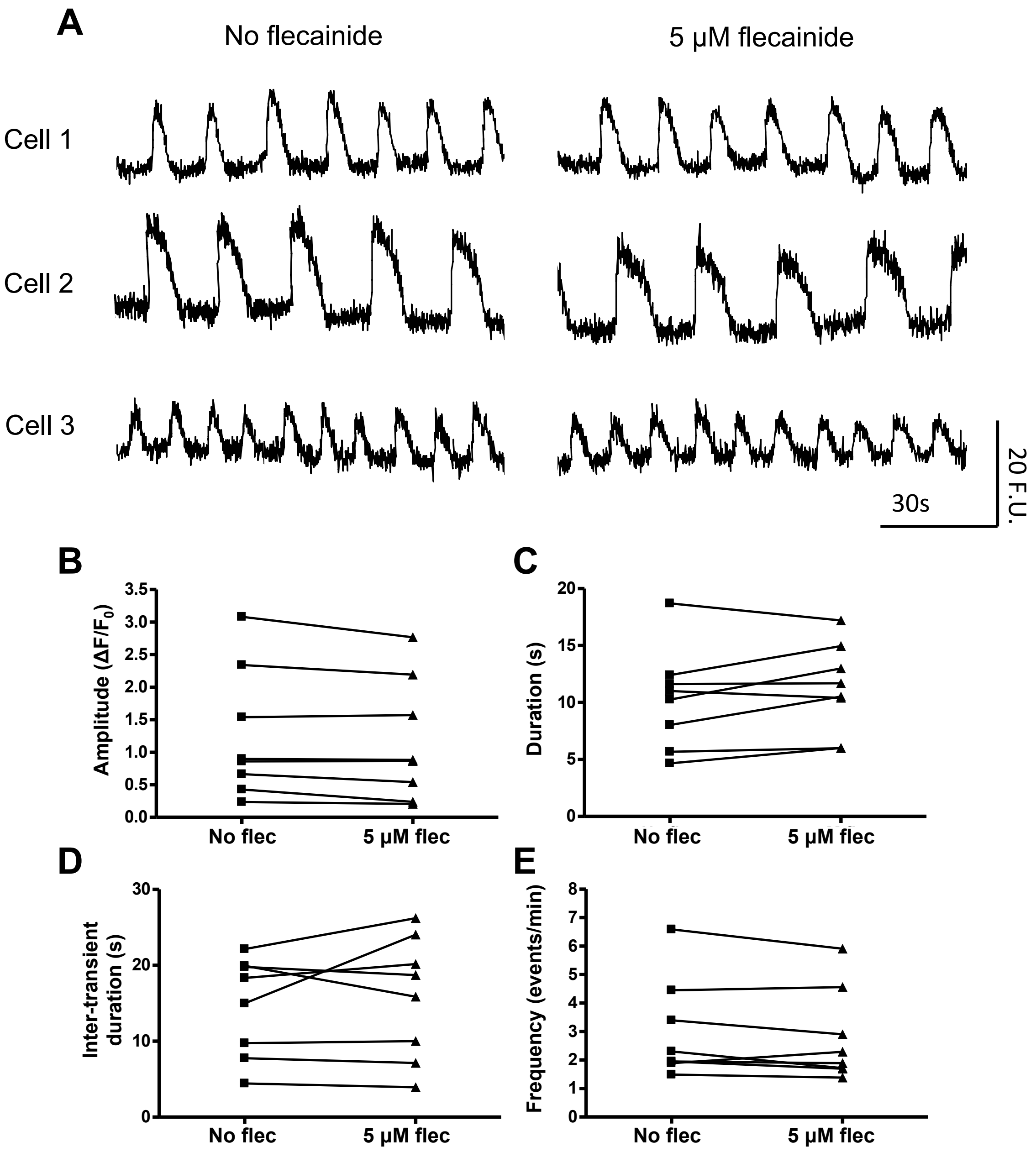


**Supplementary Figure 3.** Flecainide has no effect on spontaneous hRyR2-mediated $Ca^{2+}$ release transients in transfected HEK293 cells. **(A)** Sample traces showing spontaneous $Ca^{2+}$ release events from three different cells expressing hRyR2 in the absence and in the presence of flecainide (5 µM). Flecainide had no effect on $Ca^{2+}$ spike amplitude **(B)**, duration **(C)**, inter-transient duration **(D)** or frequency **(E)** (n=8).
